# Supplementary material for: A proton-gated channel identified in the centipede antenna
Source: EMBO Rep. 2025 Oct 20;26(24):6083–95. doi: 10.1038/s44319-025-00606-2 (PMC12714832; doi:10.1038/s44319-025-00606-2)
Supplement: Supplementary file 5 — Source data Fig. 1 [file 44319_2025_606_MOESM5_ESM.zip › Figure1F/Figure1F_colocalization_analysisi_report.pdf]

Colocalization_of_M.tif (red)_versus_M.tif (green)_4459008321.pdf

2D intensity histogram

256.0 x 256.0

Channel 2
M.tif (green)

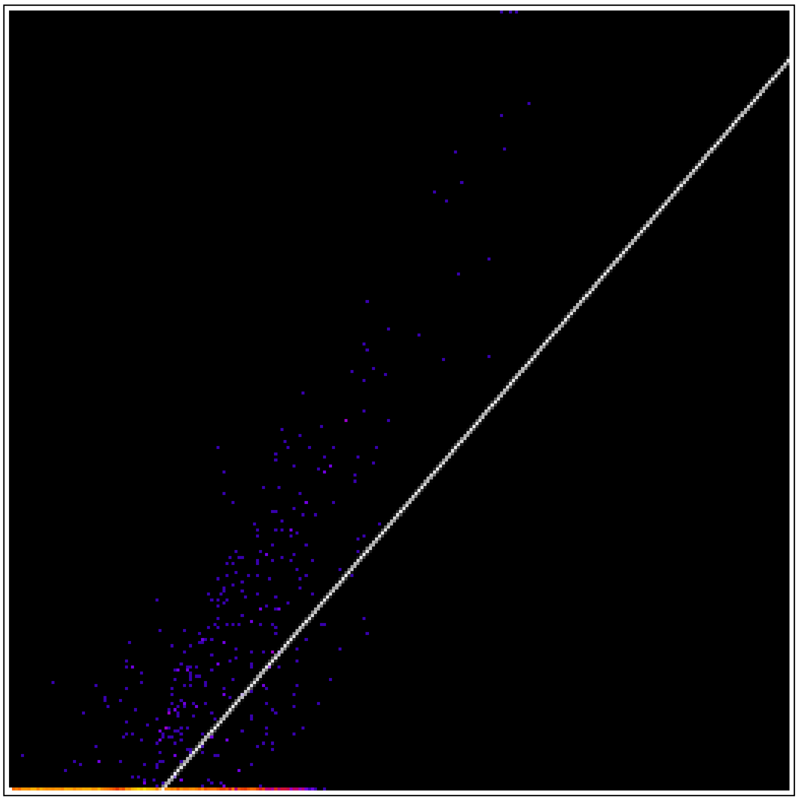

Channel 1
M.tif (red)

Smoothed & shuffled channel 1

2304.0 x 2304.0

Channel 2
M.tif (green)

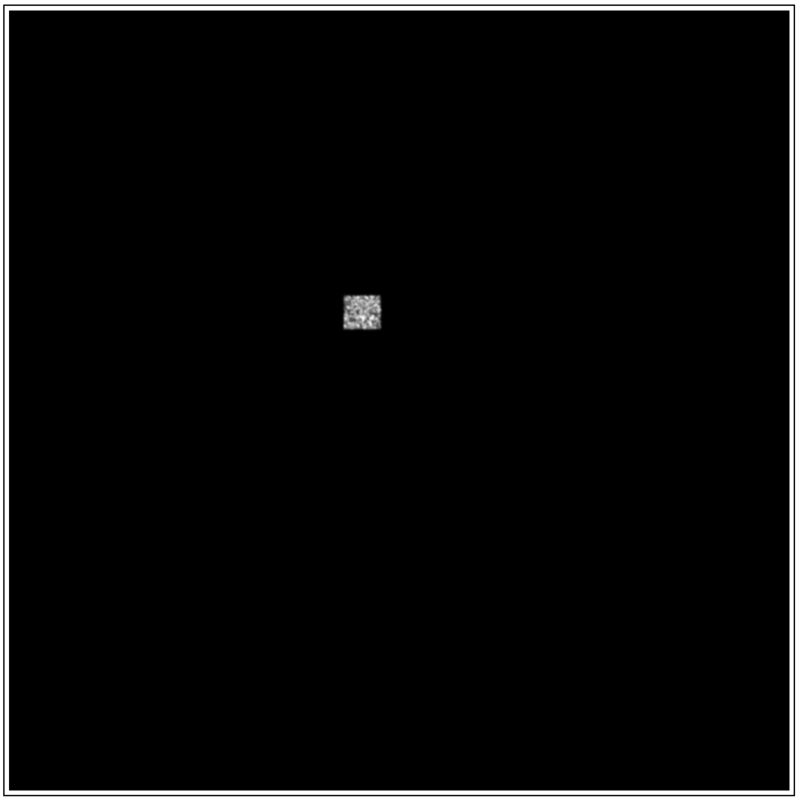

Channel 1
M.tif (red)

Channel 1 (Max Projection)

105.0 x 96.0

Channel 2
M.tif (green)

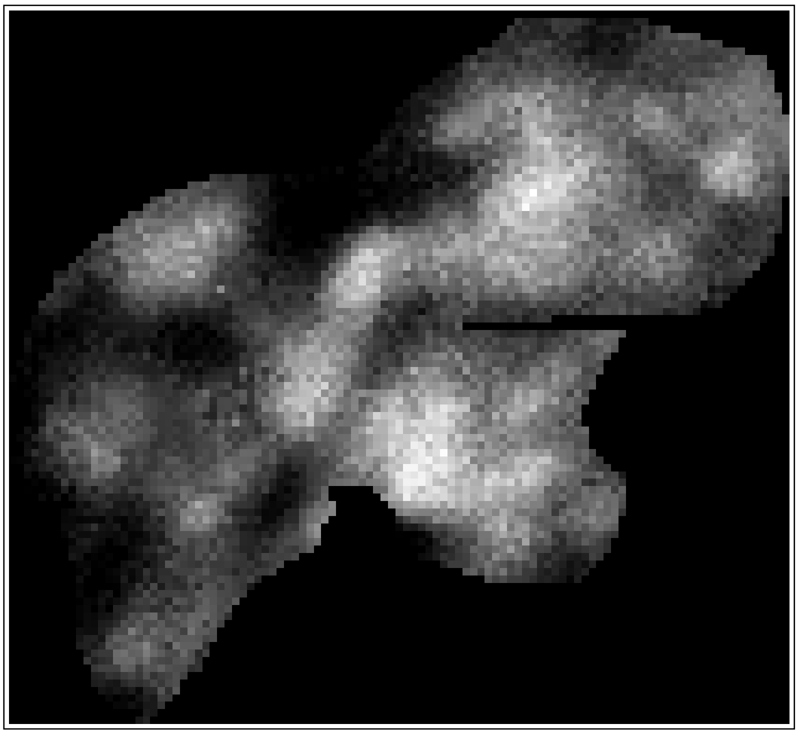

Channel 1
M.tif (red)

Channel 2 (Max Projection)

105.0 x 96.0

Channel 2
M.tif (green)

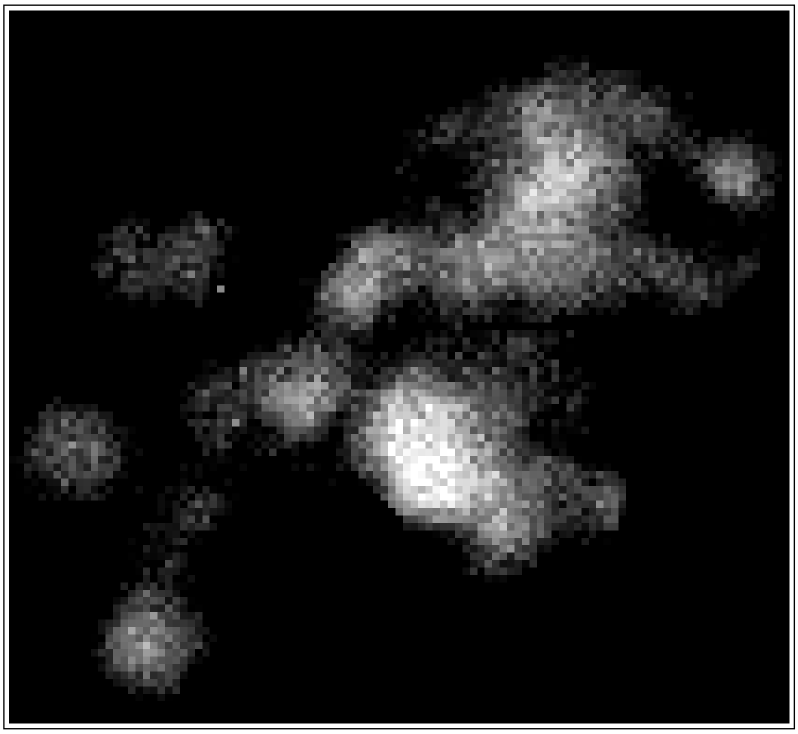

Channel 1
M.tif (red)

Warning! y-intercept far from zero - The ratio of the y-intercept of the auto threshold regression line to the mean value of Channel 2 is high. This means the y-intercept is far from zero, implying a significant positive or negative zero offset in the image data intensities. Maybe you should use a ROI. Maybe do a background subtraction in both channels. Make sure you didn't clip off the low intensities to zero. This might not affect Pearson's correlation values very much, but might harm other results.

Coloc_Job_Name: Colocalization_of_M.tif (red)_versus_M.tif (green)_445900832

% zero-zero pixels: 3.50

% saturated ch1 pixels: 0.04

% saturated ch2 pixels: 0.97

Channel 1 Max: 191.000

Channel 2 Max: 255.000

Channel 1 Min: 0.000

Channel 2 Min: 0.000

Channel 1 Mean: 64.423

Channel 2 Mean: 42.184

Channel 1 Integrated (Sum) Intensity: 356970.000

Channel 2 Integrated (Sum) Intensity: 233741.000

Mask Type Used: mask image

Mask ID Used: 445900832

m (slope): 1.56

b (y-intercept): -58.47

b to y-mean ratio: -1.39

Ch1 Max Threshold: 46.00

Ch2 Max Threshold: 14.00

Threshold regression: Costes

Pearson's R value (no threshold): 0.81

Pearson's R value (below threshold): -0.00

Pearson's R value (above threshold): 0.78

Spearman's rank correlation value: 0.76531743

Spearman's correlation t-statistic: 88.4923

t-statistic degrees of freedom: 5539.000

Manders' M1 (Above zero intensity of Ch2): 0.749

Manders' M2 (Above zero intensity of Ch1): 1.000

Manders' tM1 (Above autothreshold of Ch2): 0.705

Manders' tM2 (Above autothreshold of Ch1): 0.961

Kendall's Tau-b rank correlation value: 0.6081

Costes P-Value: 1.00

Costes Shuffled Mean: 0.06

Costes Shuffled Std.D.: 0.12

Ratio of rand. Pearsons >= actual Pearsons value : 0.00
