# Supplementary figures and images for: A proton-gated channel identified in the centipede antenna
Source: EMBO Rep. 2025 Oct 20;26(24):6083–95. doi: 10.1038/s44319-025-00606-2 (PMC12714832; doi:10.1038/s44319-025-00606-2)

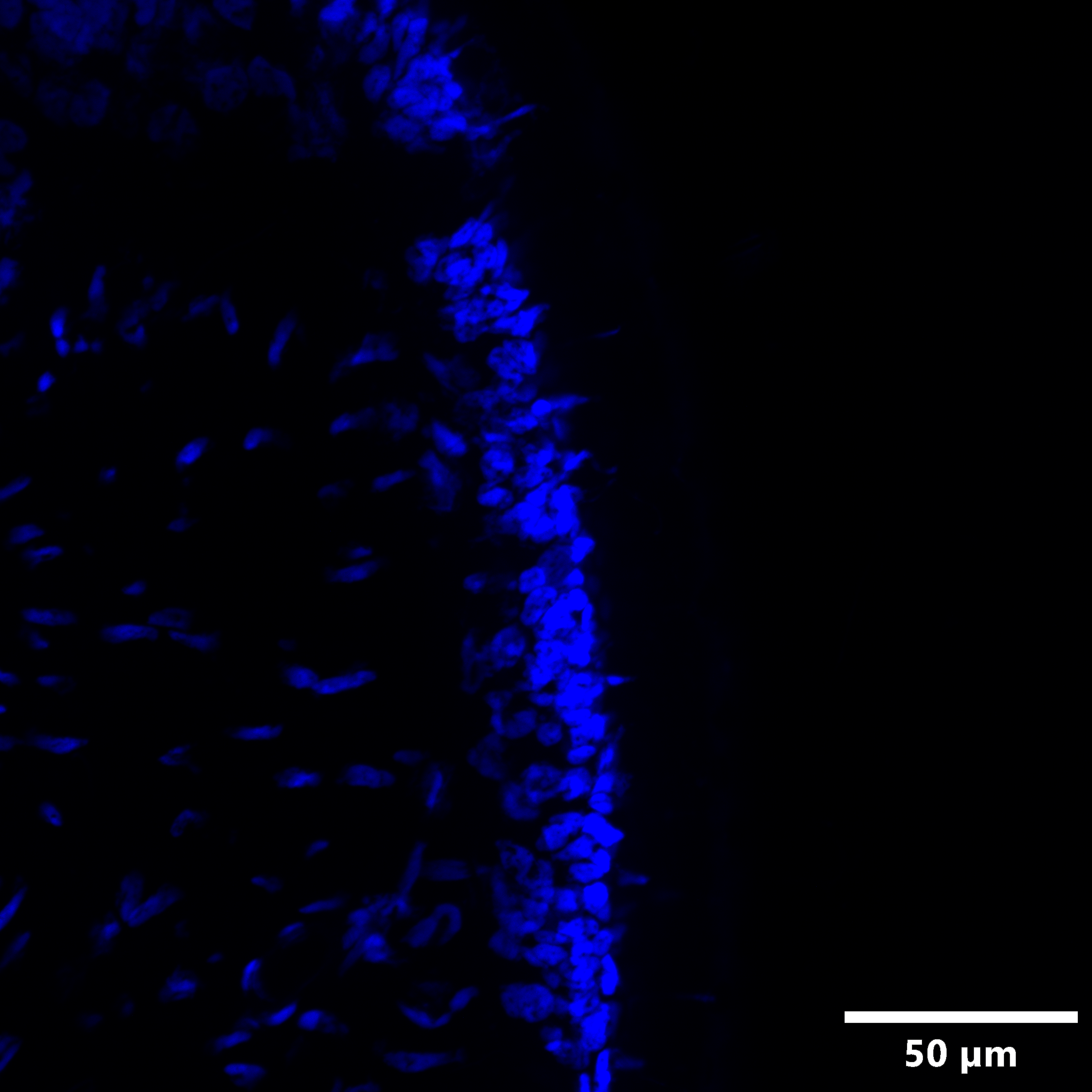

Supplement: Supplementary file 5 — Source data Fig. 1 [file 44319_2025_606_MOESM5_ESM.zip › Figure1E/Blue_DAPI.tif]

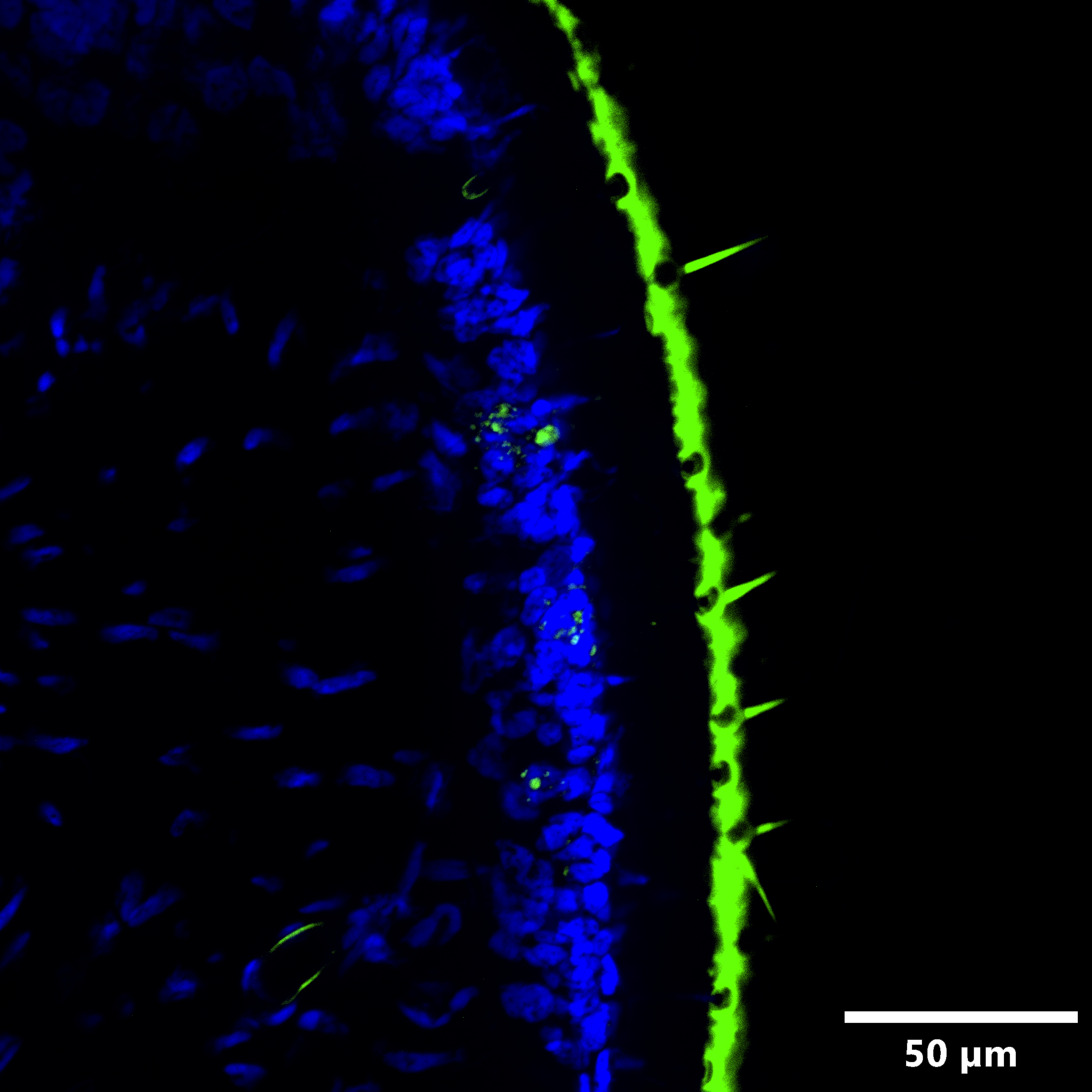

Supplement: Supplementary file 5 — Source data Fig. 1 [file 44319_2025_606_MOESM5_ESM.zip › Figure1E/Green_PDPNaC1.tif]

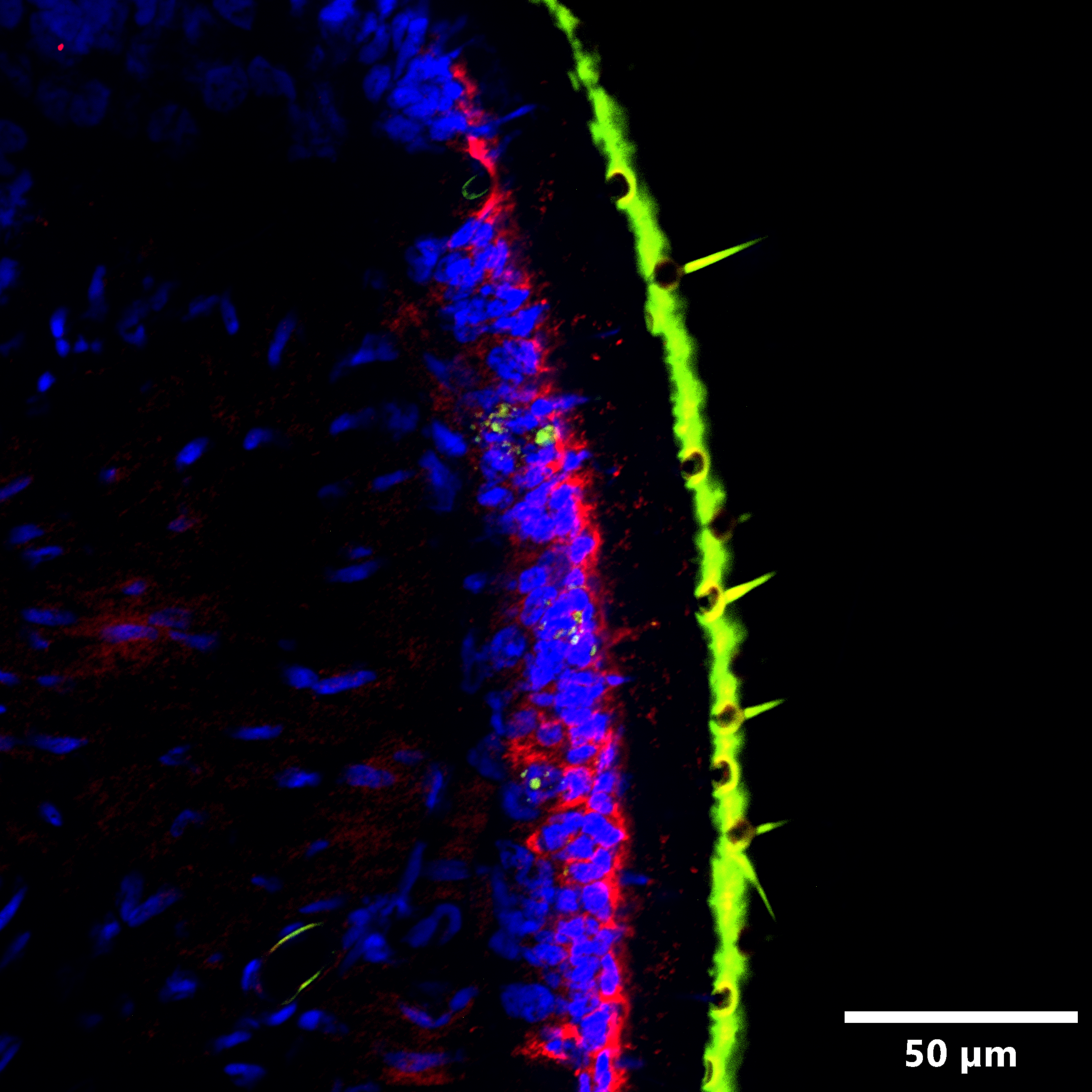

Supplement: Supplementary file 5 — Source data Fig. 1 [file 44319_2025_606_MOESM5_ESM.zip › Figure1E/Merge.tif]

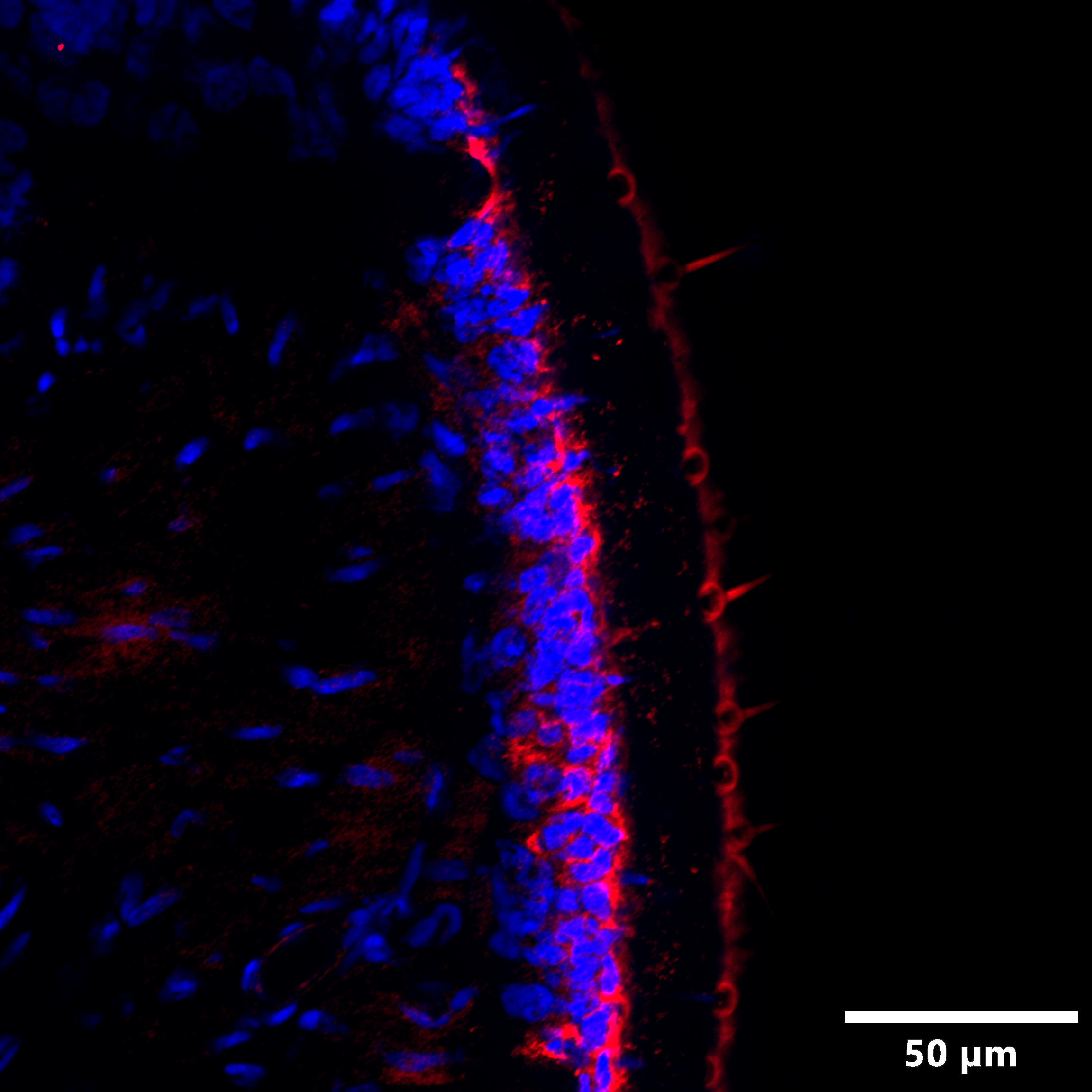

Supplement: Supplementary file 5 — Source data Fig. 1 [file 44319_2025_606_MOESM5_ESM.zip › Figure1E/Red_PIEZO2.tif]

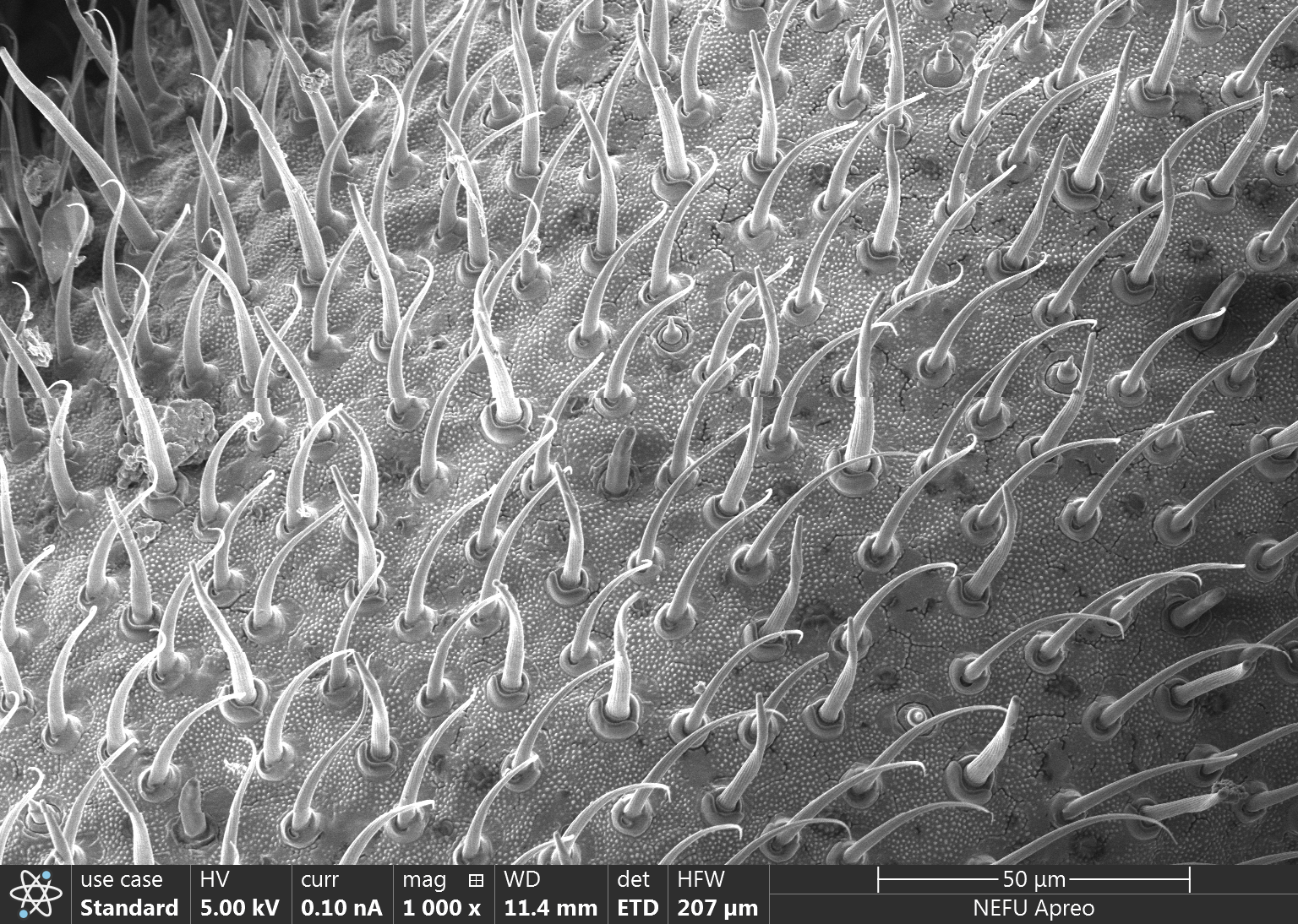

Supplement: Supplementary file 5 — Source data Fig. 1 [file 44319_2025_606_MOESM5_ESM.zip › Figure1G/Figure1G_SEM_original_images.tif]

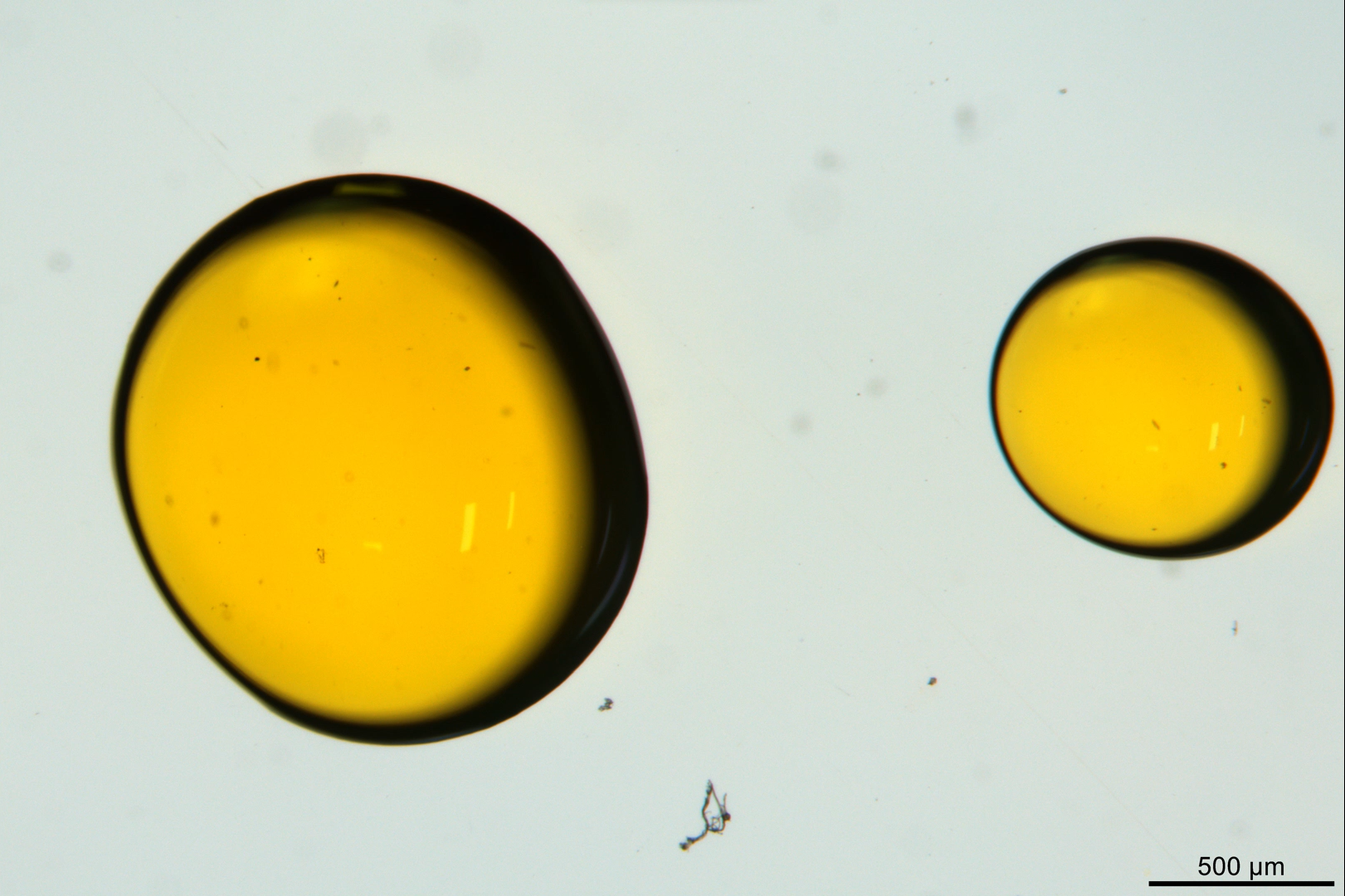

Supplement: Supplementary file 5 — Source data Fig. 1 [file 44319_2025_606_MOESM5_ESM.zip › Figure1H/FIG 1H_1.png]

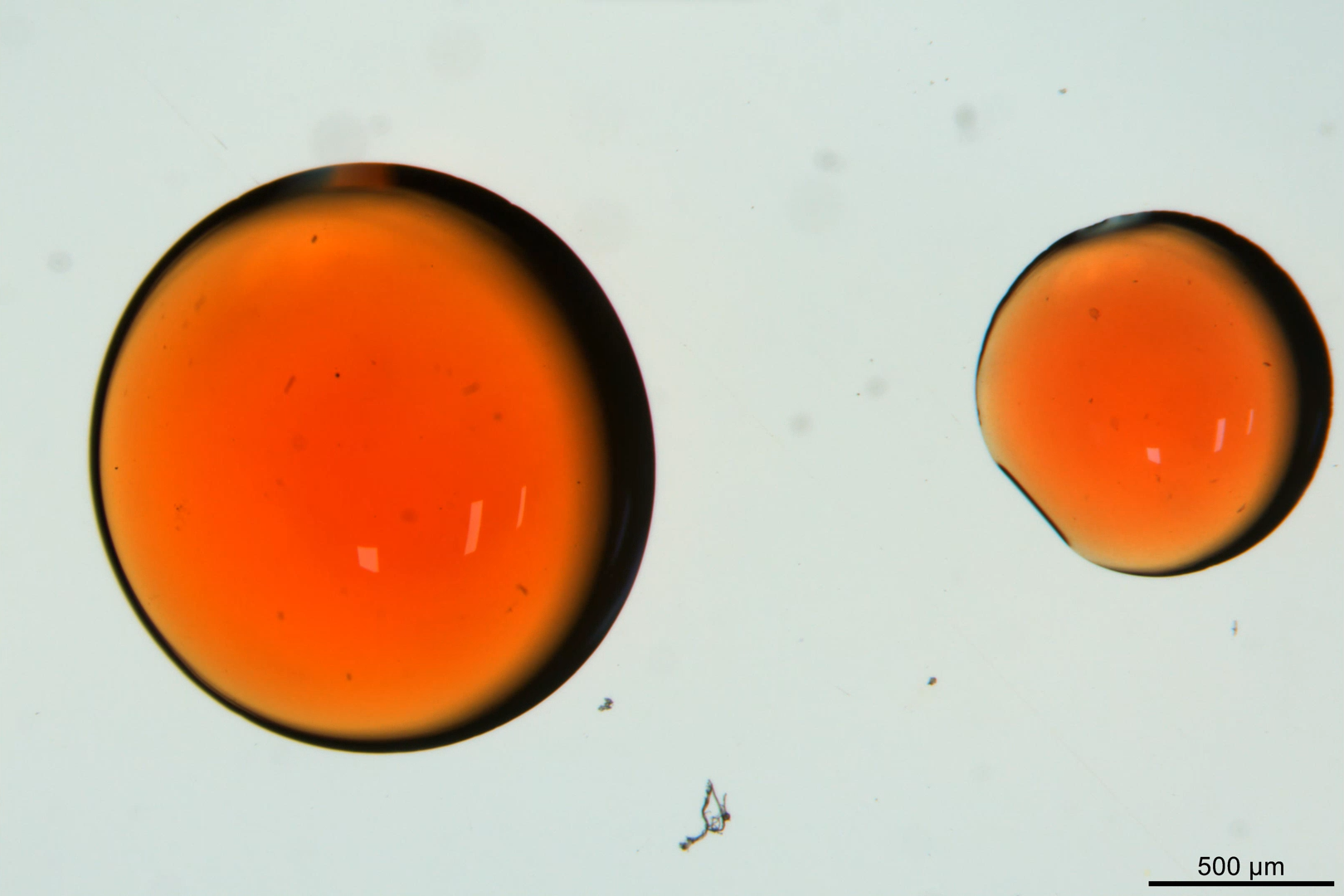

Supplement: Supplementary file 5 — Source data Fig. 1 [file 44319_2025_606_MOESM5_ESM.zip › Figure1H/FIG 1H_2.png]

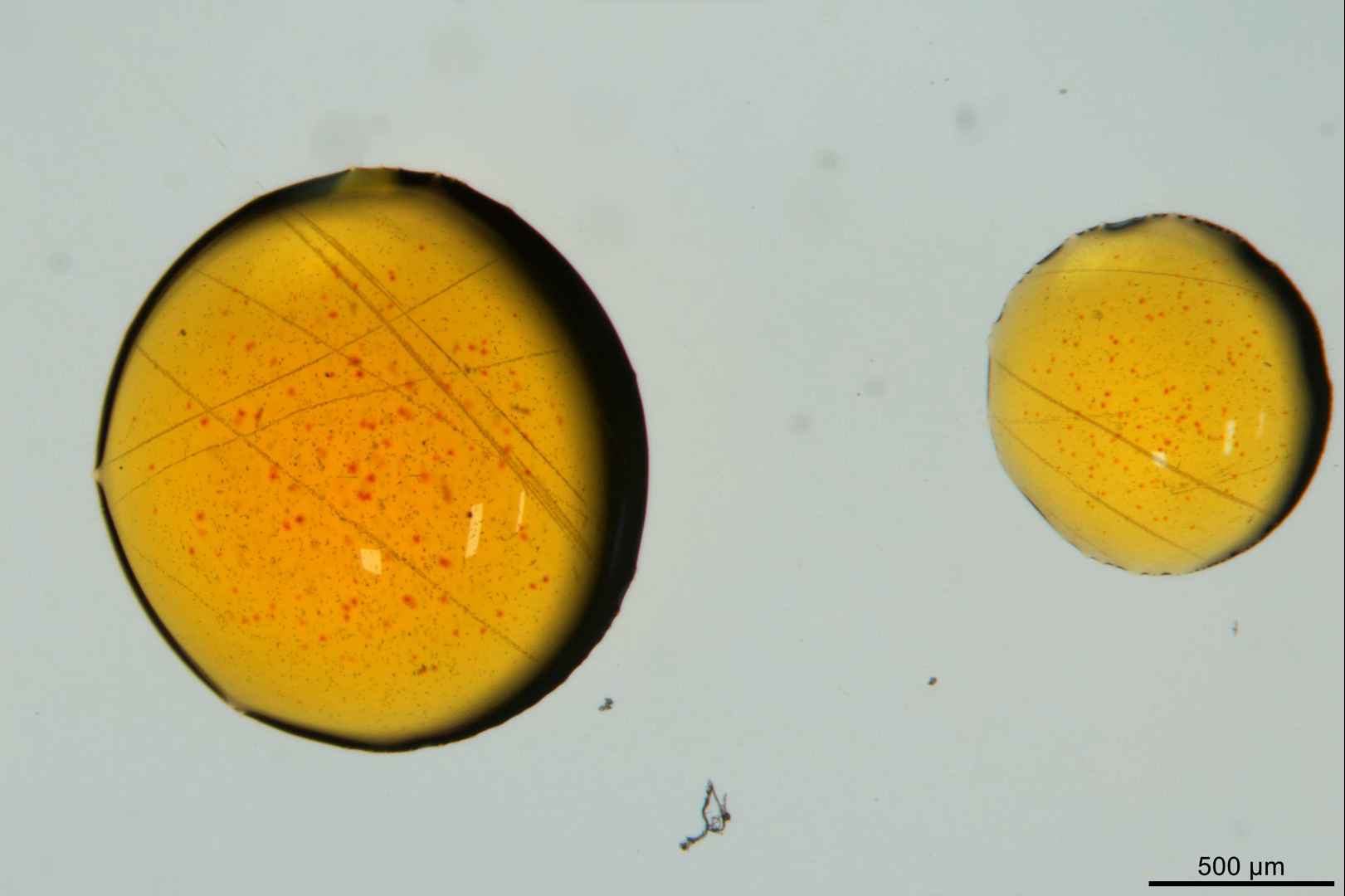

Supplement: Supplementary file 5 — Source data Fig. 1 [file 44319_2025_606_MOESM5_ESM.zip › Figure1H/FIG 1H_3.png]

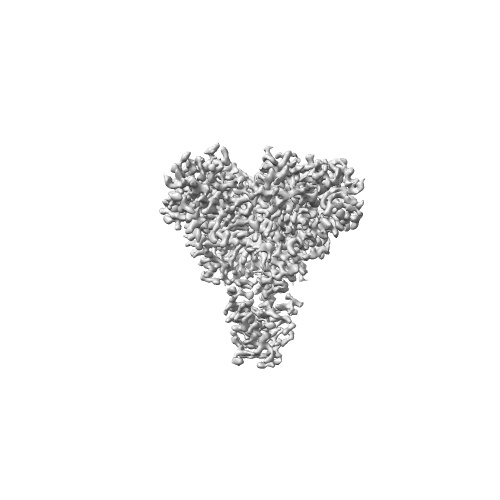

Supplement: Supplementary file 7 — Source data Fig. 3 [file 44319_2025_606_MOESM7_ESM.zip › Figure3A_and_B/9JF7.jpg]

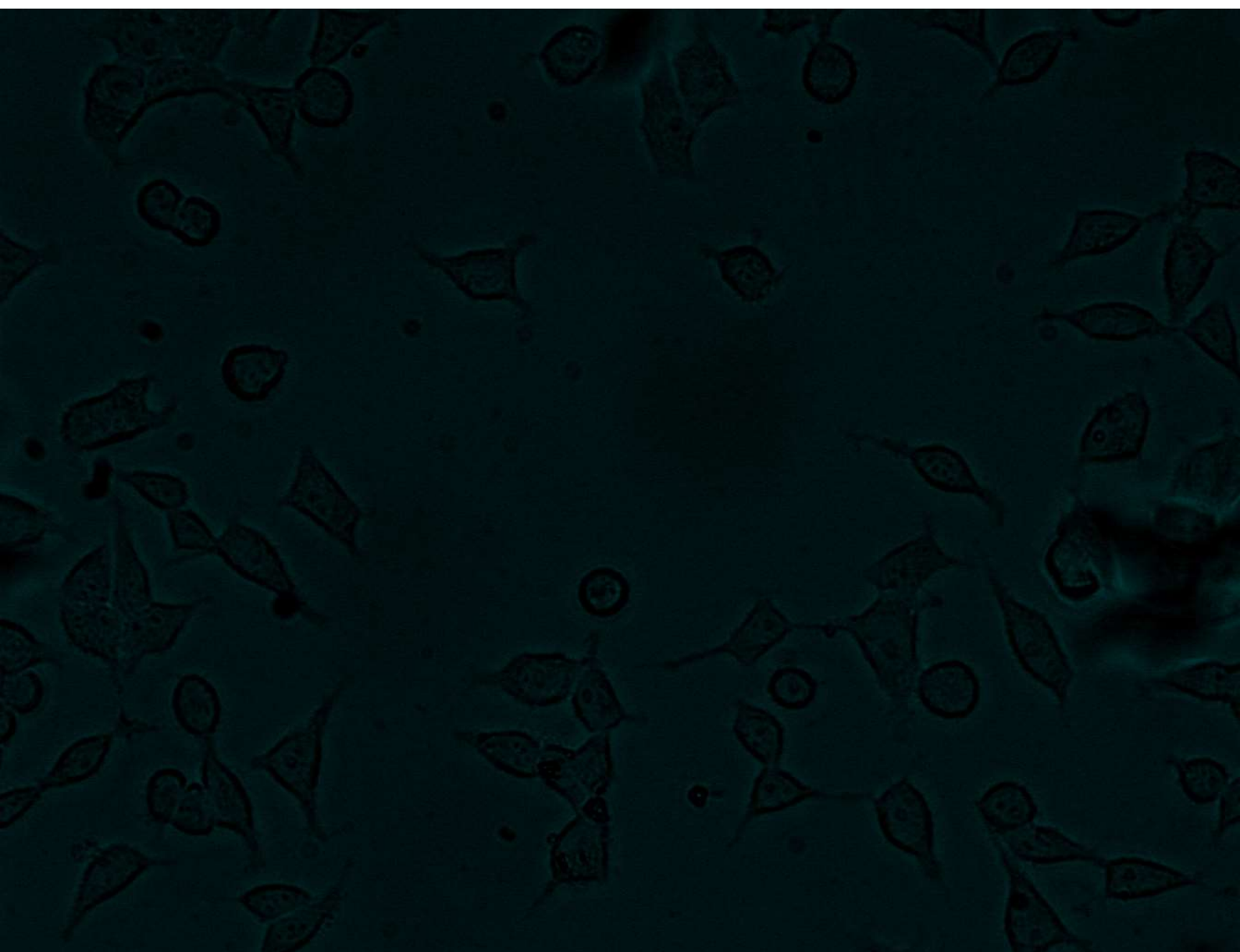

Supplement: Supplementary file 9 — Source data Fig. 5 [file 44319_2025_606_MOESM9_ESM.zip › Figure5C/Figure5C_ANAP_negtive_cell_fluorescence_image.pdf]

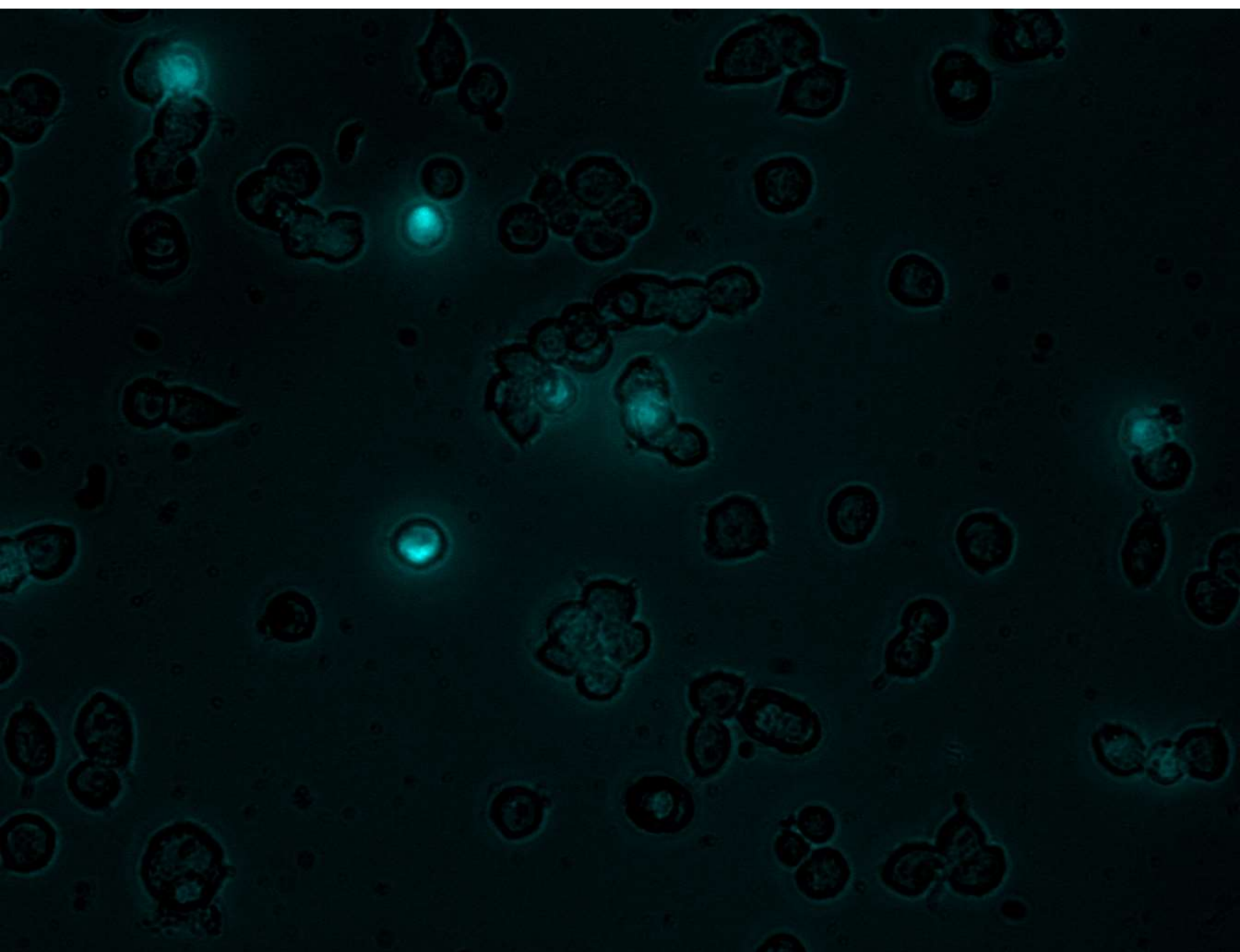

Supplement: Supplementary file 9 — Source data Fig. 5 [file 44319_2025_606_MOESM9_ESM.zip › Figure5C/Figure5C_ANAP_positive_cell_fluorescence_image.pdf]

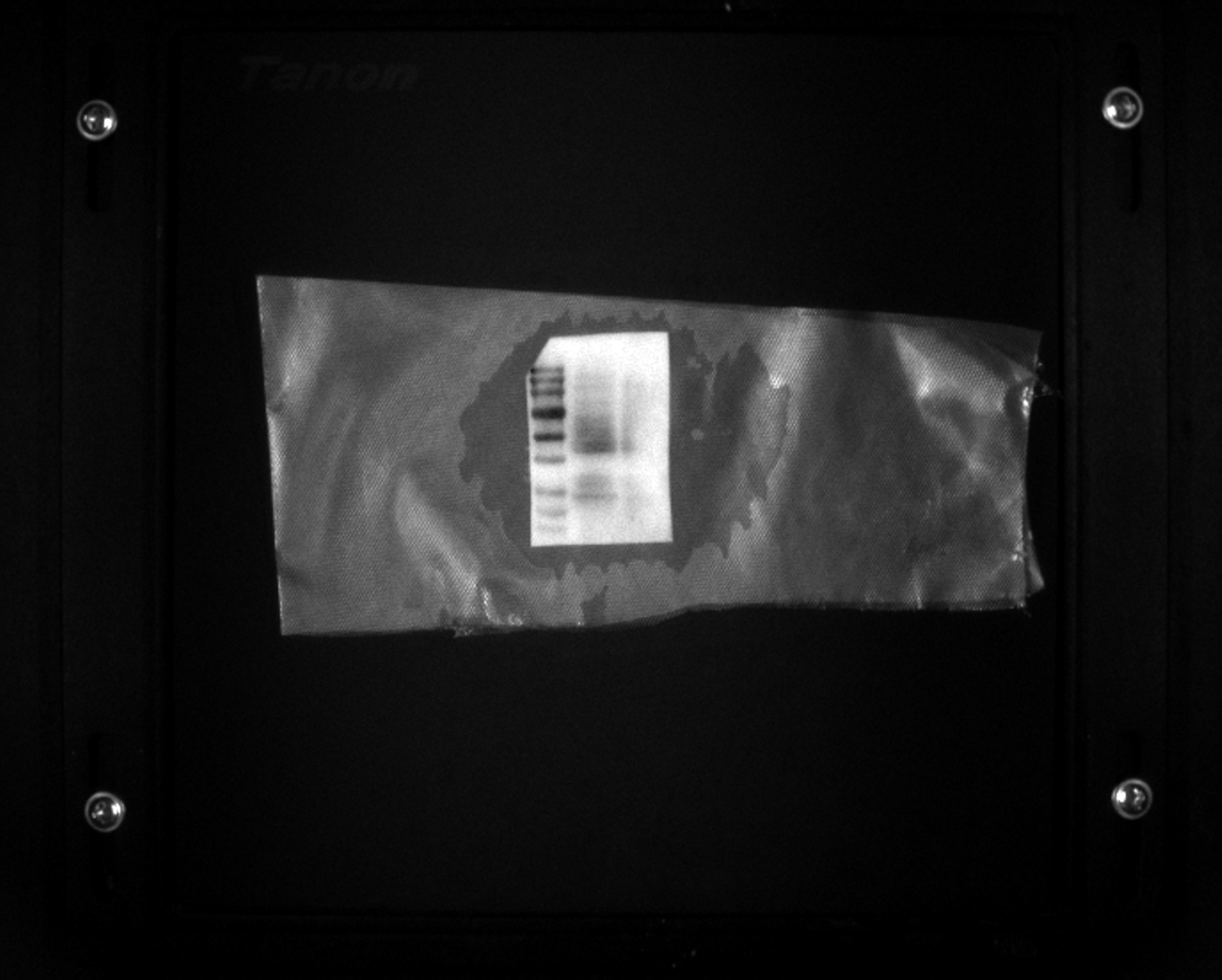

Supplement: Supplementary file 10 — SourceDataForAppendix [file 44319_2025_606_MOESM10_ESM.zip › EMBOR-2025-62006V2_SourceDataForAppendix/AppendixFigS13/AppendixFigS13A/AppendixFigS13A_western_PDPNaC1.Tif]

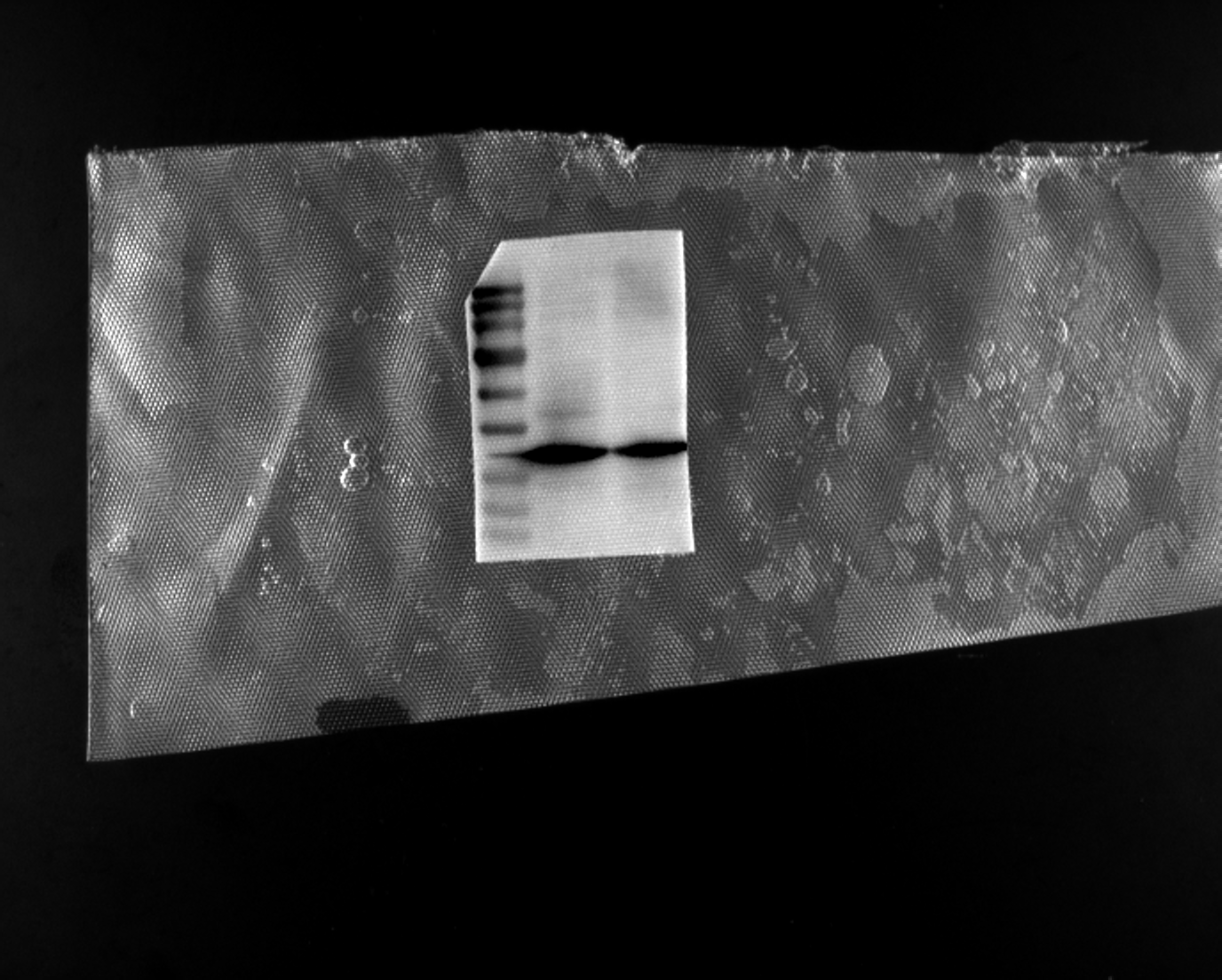

Supplement: Supplementary file 10 — SourceDataForAppendix [file 44319_2025_606_MOESM10_ESM.zip › EMBOR-2025-62006V2_SourceDataForAppendix/AppendixFigS13/AppendixFigS13A/AppendixFigS13A_western_PDPNaC1_GAPDH.tif]

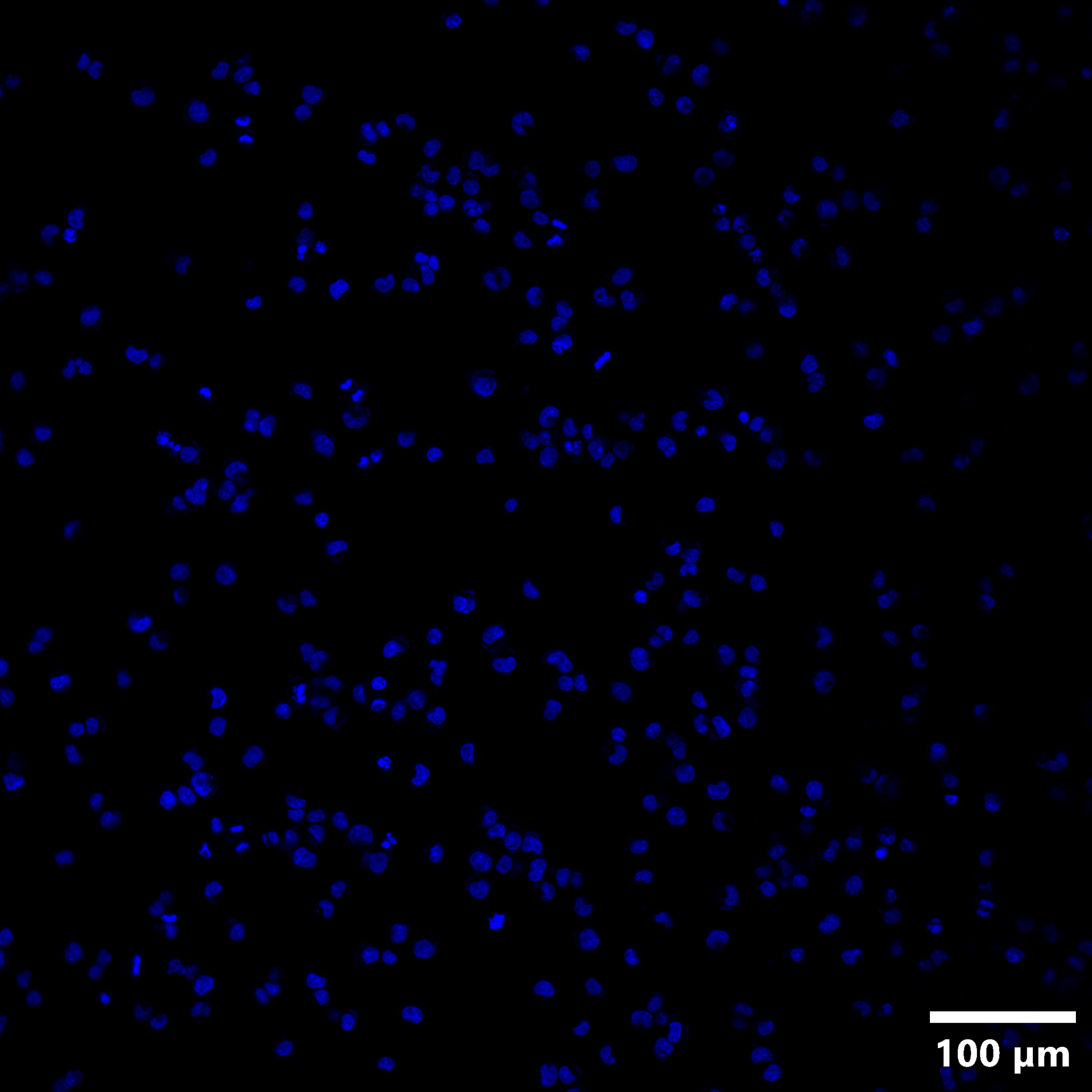

Supplement: Supplementary file 10 — SourceDataForAppendix [file 44319_2025_606_MOESM10_ESM.zip › EMBOR-2025-62006V2_SourceDataForAppendix/AppendixFigS13/AppendixFigS13B/AppendixFigS13B_IF_DAPI.tif]

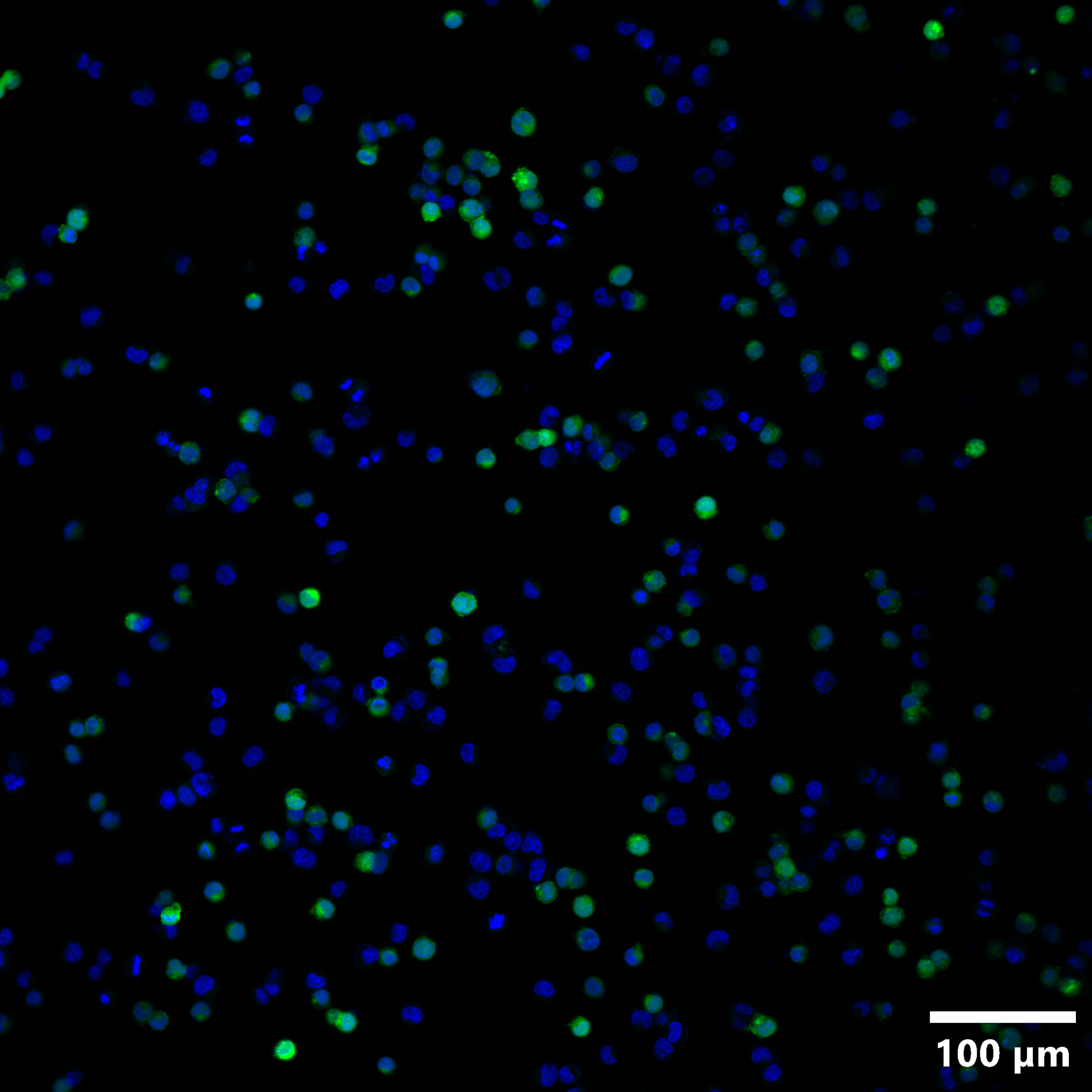

Supplement: Supplementary file 10 — SourceDataForAppendix [file 44319_2025_606_MOESM10_ESM.zip › EMBOR-2025-62006V2_SourceDataForAppendix/AppendixFigS13/AppendixFigS13B/AppendixFigS13B_IF_Merge.tif]

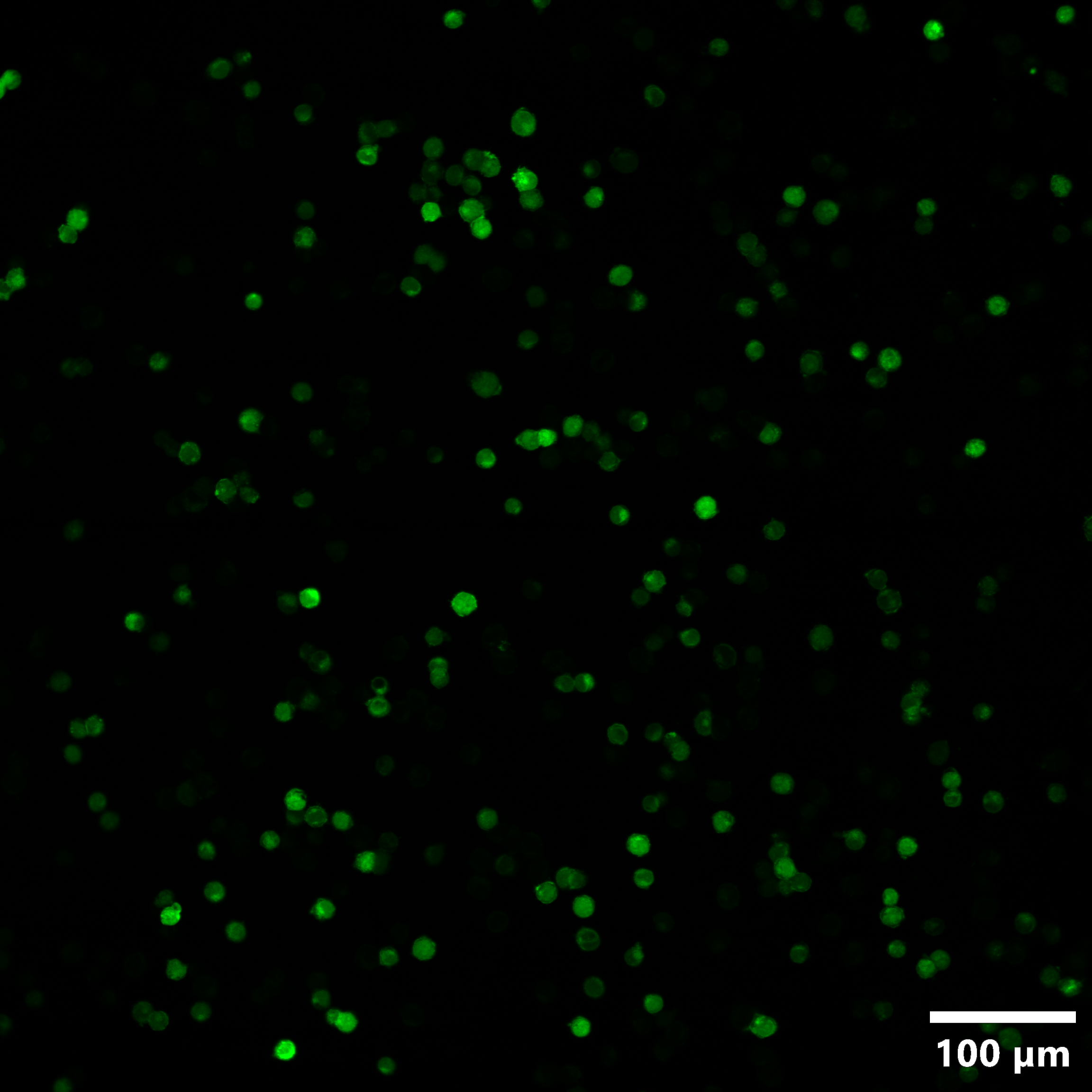

Supplement: Supplementary file 10 — SourceDataForAppendix [file 44319_2025_606_MOESM10_ESM.zip › EMBOR-2025-62006V2_SourceDataForAppendix/AppendixFigS13/AppendixFigS13B/AppendixFigS13B_IF_PDPNaC1.tif]

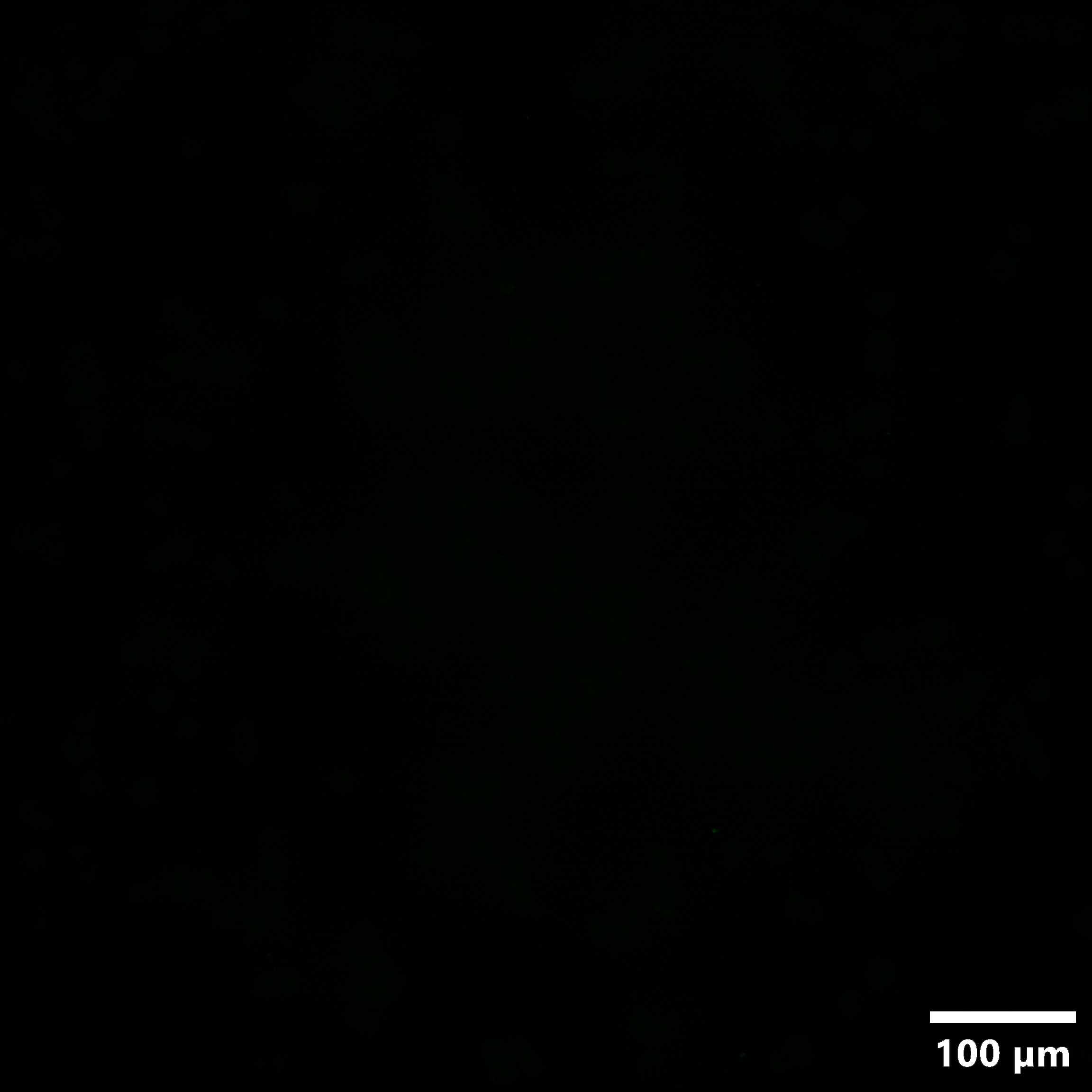

Supplement: Supplementary file 10 — SourceDataForAppendix [file 44319_2025_606_MOESM10_ESM.zip › EMBOR-2025-62006V2_SourceDataForAppendix/AppendixFigS13/AppendixFigS13B/AppendixFigS13B_IF_untransfected control.tif]

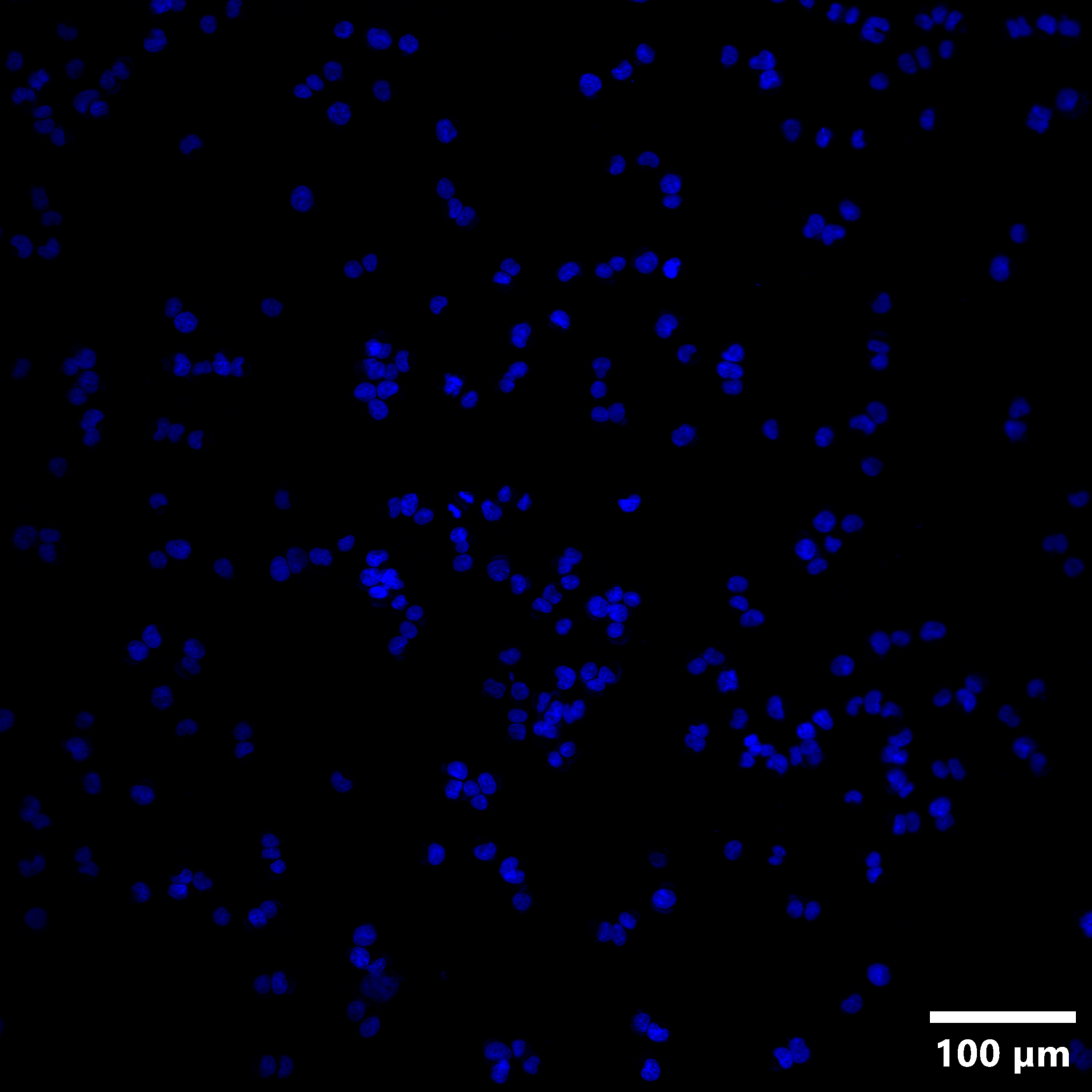

Supplement: Supplementary file 10 — SourceDataForAppendix [file 44319_2025_606_MOESM10_ESM.zip › EMBOR-2025-62006V2_SourceDataForAppendix/AppendixFigS13/AppendixFigS13B/AppendixFigS13B_IF_untransfected control_DAPI.tif]

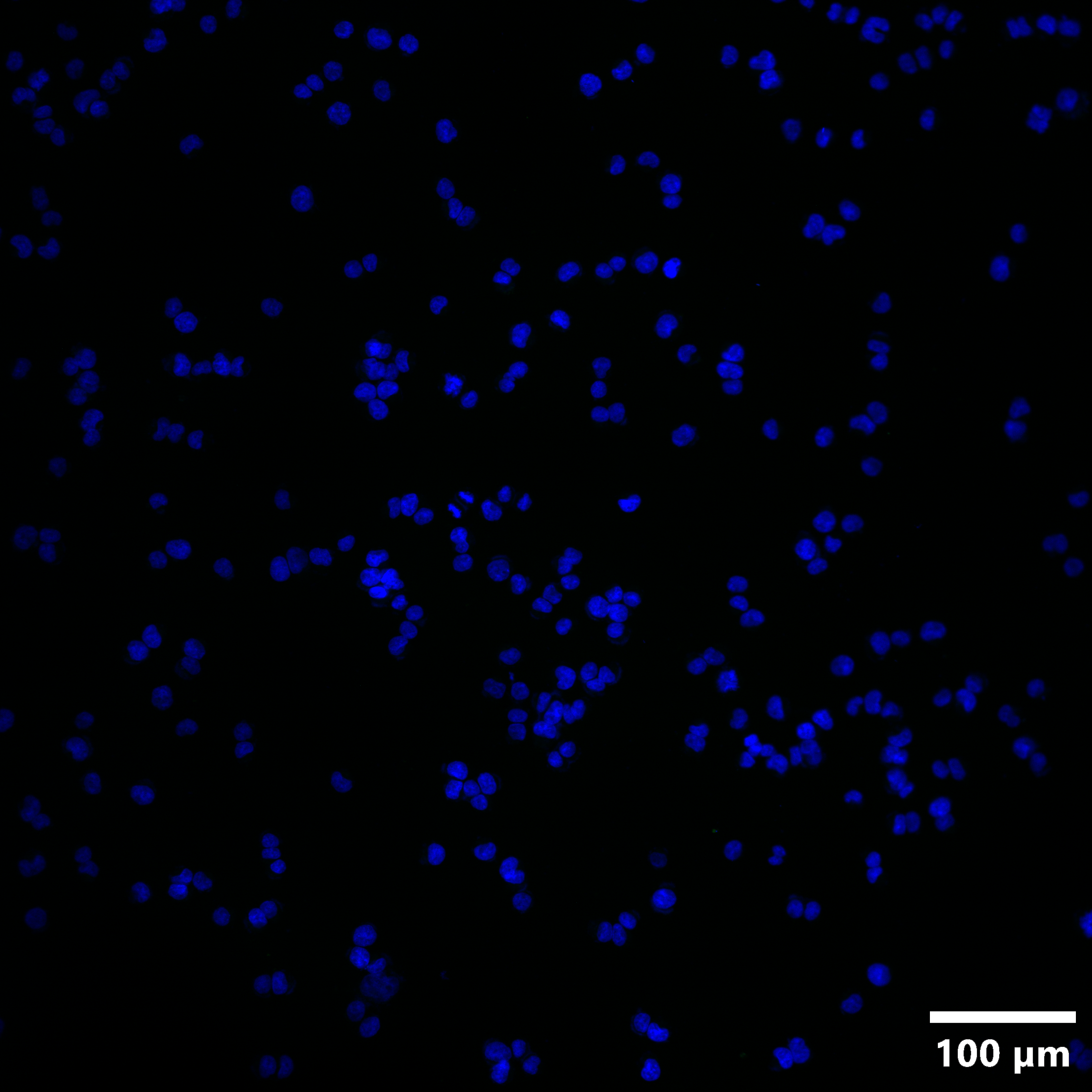

Supplement: Supplementary file 10 — SourceDataForAppendix [file 44319_2025_606_MOESM10_ESM.zip › EMBOR-2025-62006V2_SourceDataForAppendix/AppendixFigS13/AppendixFigS13B/AppendixFigS13B_IF_untransfected control_Merge.tif]

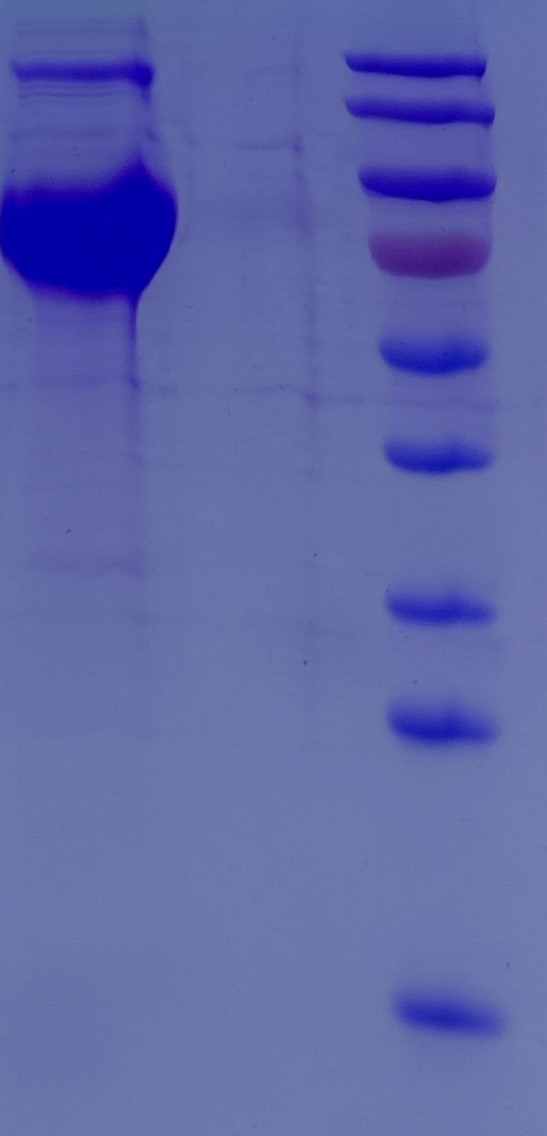

Supplement: Supplementary file 10 — SourceDataForAppendix [file 44319_2025_606_MOESM10_ESM.zip › EMBOR-2025-62006V2_SourceDataForAppendix/AppendixFigS6/AppendixFigS6A/AppendixFigS6A_western_blot.jpg]
