# Supplementary material for: A proton-gated channel identified in the centipede antenna
Source: EMBO Rep. 2025 Oct 20;26(24):6083–95. doi: 10.1038/s44319-025-00606-2 (PMC12714832; doi:10.1038/s44319-025-00606-2)
Supplement: Supplementary file 1 — Appendix [file 44319_2025_606_MOESM1_ESM.pdf]

APPENDIX

A Proton-Gated Channel Identified in the Centipede Antenna

Wenqi Dong, Licheng Yuan, Jiangming Shang, Fan Yang, Shilong Yang, Xiancui Lu, Qian Wang, Anna Luo, Jiheng Geng, Jiatong Cheng, Runze Li, Yunfei Wang*

* Correspondence: wangyunfei@nefu.edu.cn

TABLE OF CONTENTS

Appendix Figures

Appendix Figure S1. Isoform 151426 belongs to the DEG/ENaC family. 2
Appendix Figure S2. UMAP plot. 3
Appendix Figure S3. Relationship between droplet diameter and pH transition kinetics. 4
Appendix Figure S4. Calcium-dependent regulation of PDPNaC1 proton sensitivity. 5
Appendix Figure S5. Ion permeabilities for cASIC1a. 6
Appendix Figure S6. Protein collection and structure determination of PDPNaC1. 7
Appendix Figure S7. Ribbon diagram of single subunit of PDPNaC1. 8
Appendix Figure S8. Sequence alignment. 9
Appendix Figure S9. Ion selectivity of PDPNaC1 and mutants. 10
Appendix Figure S10. Functional analysis of ANAP-incorporated mutants. 11
Appendix Figure S11. Representative emission spectra of ANAP under different pH and DMSO conditions. 12
Appendix Figure S12. Electrostatic potential map of PDPNaC1. 13
Appendix Figure S13. Anti-PDPNaC1 antibody validation by western blot and immunofluorescence in cultured cells. 14
Appendix Figure S14. Representative current traces of PDPNaC1-expressing cells, showing the presence (A) or absence (B) of detectable endogenous ASIC currents. 15

Appendix Tables

Appendix Table S1. Cell number and annotation for each cell cluster and literature references for marker genes... 16
Appendix Table S2. List of proteins used for sequence alignment in this article. 17
Appendix Table S3. Primers used in this study to generate single-point mutants. 20
Appendix Table S4. Data collection and refinement statistics. 21

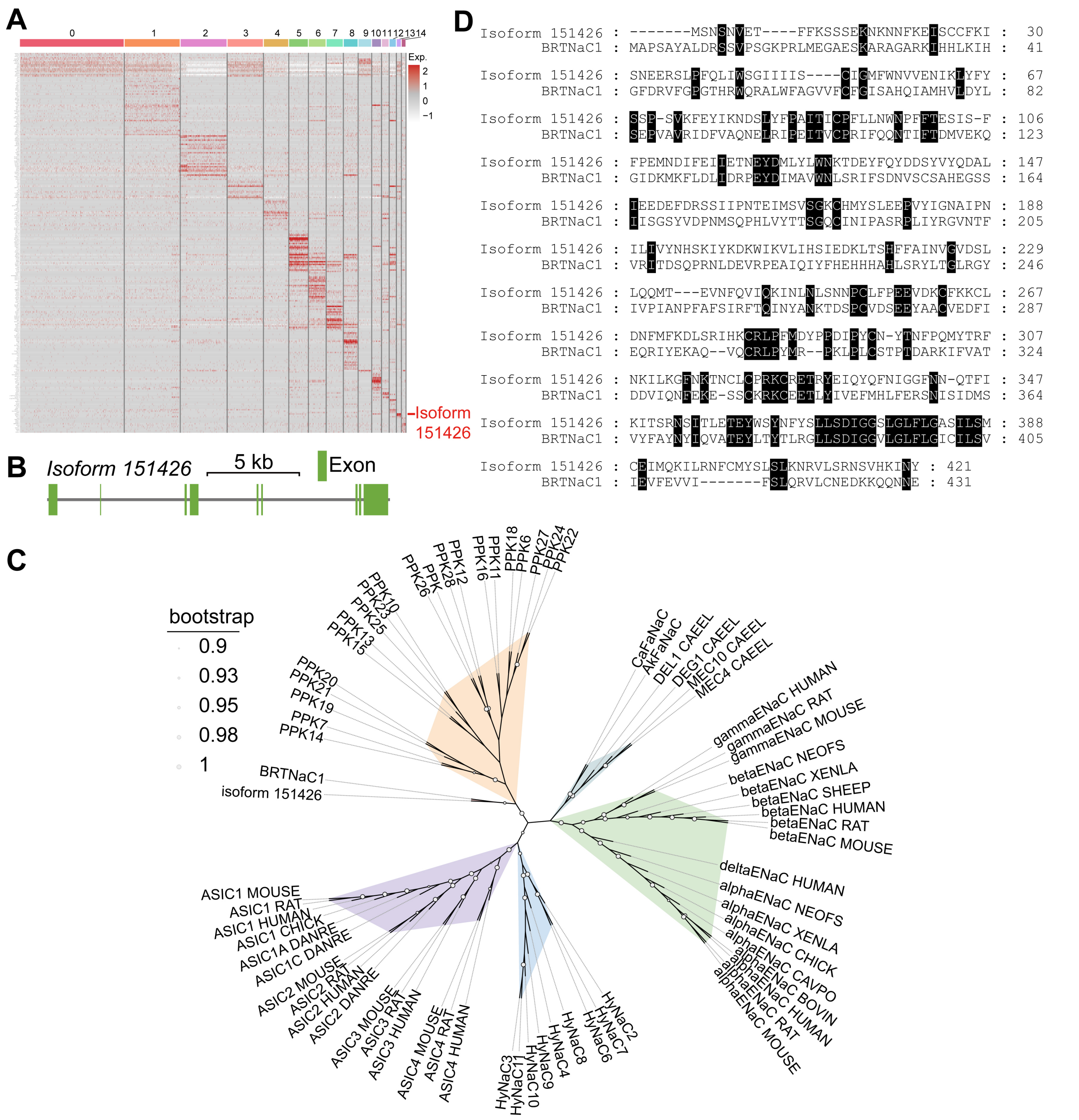

Appendix Figure S1. Isoform 151426 belongs to the DEG/ENaC family.

(A) Heatmaps illustrating differential gene expression patterns in each neuronal cell subcluster.

(B) Schematic diagram of the intron-exon structure of the *isoform 151426* gene.

(C) Phylogenetic tree of EDG/ENaC proteins and isoform 151426. Multiple sequence alignment was performed using the MUSCLE algorithm implemented in MEGA X software (v 10.2.6). The phylogenetic tree was generated using the Neighbor-Joining method. Numbers at the nodes represent bootstrap percentages from 1,000 replications (for Gene IDs see Appendix Table S2). The resulting tree was visualized and annotated using the iTOL online tool (Interactive Tree Of Life, https://itol.embl.de).

(D) Protein sequence alignment of isoform 151426 and BRTNaC1 (for Gene ID see Appendix Table S2), with identical residues highlighted in black.

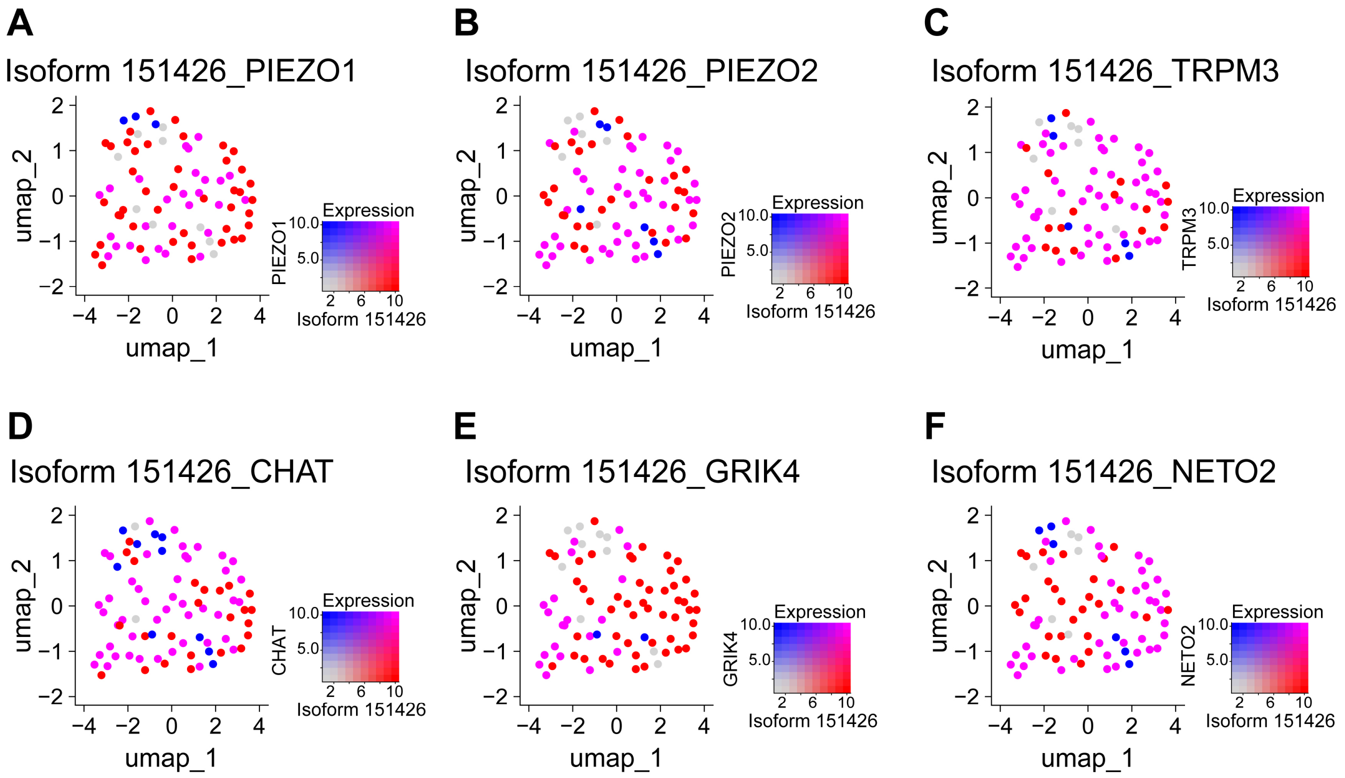

Appendix Figure S2. UMAP plot.

UMAP plot showing the expression of *isoform 151426* and genes related to excitatory neurons in neuronal subcluster 13.

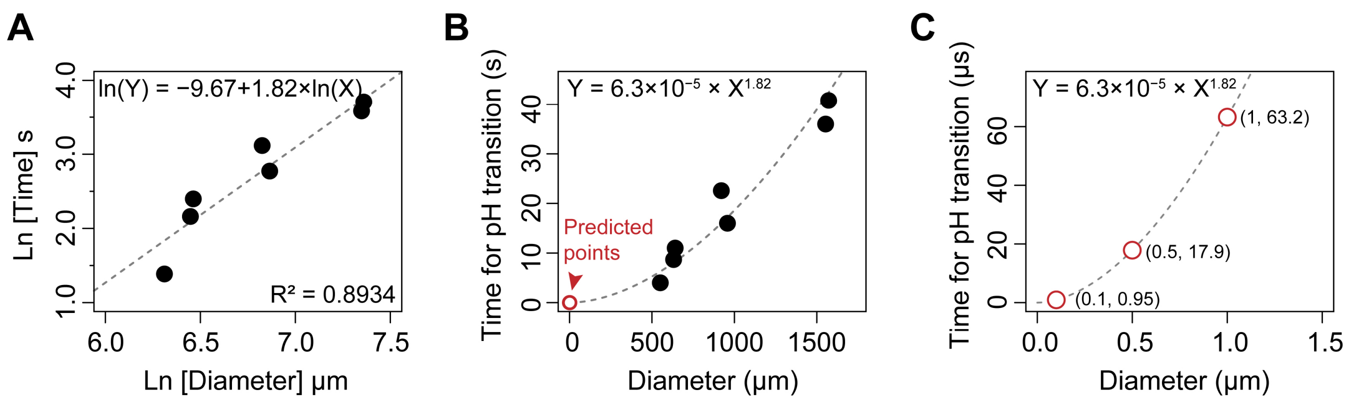

Appendix Figure S3. Relationship between droplet diameter and pH transition kinetics.

(A) Natural logarithmic transformation (ln-ln plot) of droplet diameter versus pH transition time (from pH 3 to pH 7) with linear regression fit ($R^2 = 0.8934$).

(B) Power-law function ($Y = A \times X^b$) derived from the linear fit in (A), showing original data points (black) and predicted values for 0.1, 0.5, and 1 μm droplets (red).

(C) Magnified view of predicted points from (B) with coordinate annotations.

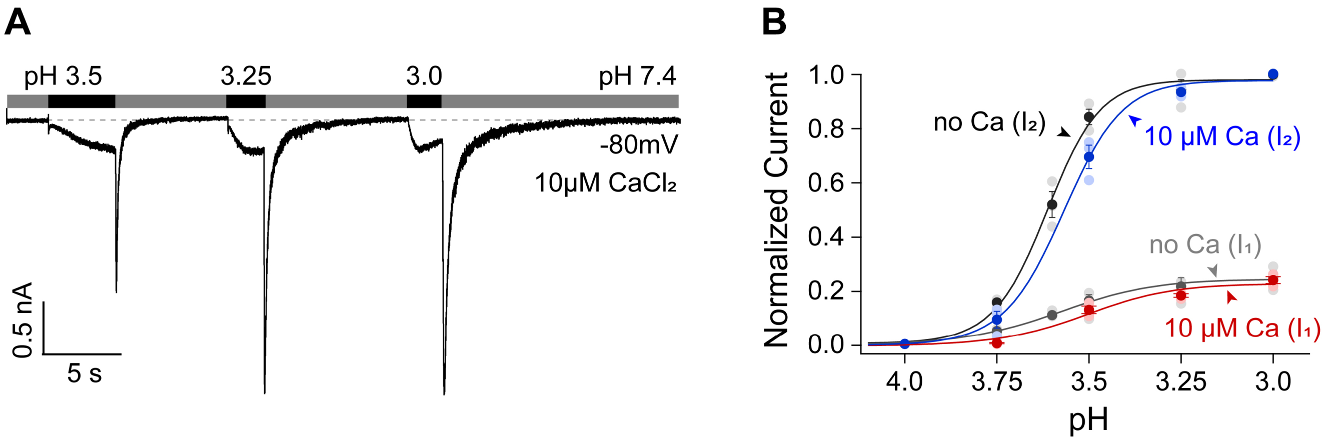

Appendix Figure S4. Calcium-dependent regulation of PDPNaC1 proton sensitivity.

(A) Representative current traces of PDPNaC1-expressing cells under different pH conditions, all containing 10 μM $CaCl_2$ (V_m = -80 mV).

(B) Dose-response curves of PDPNaC1 activated by proton in the absence and presence of 10 μM $CaCl_2$ (*n* = 3 cells for each group). Data represent mean ± SEM fitted with the Hill equation. Currents were normalized to the maximum amplitude.

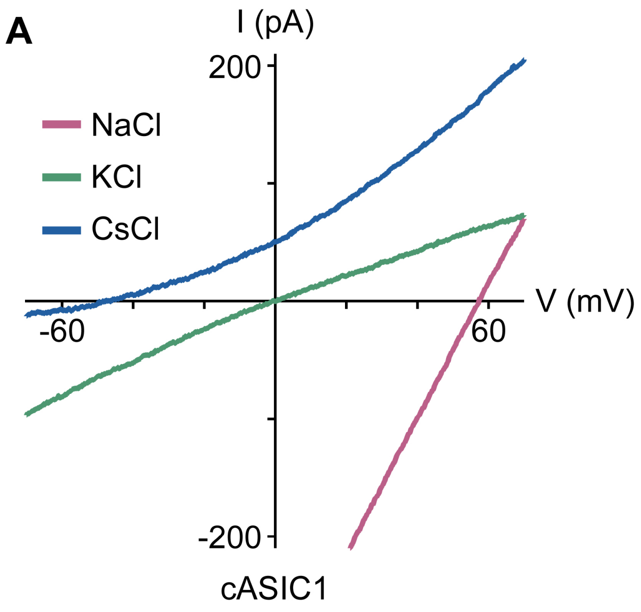

Appendix Figure S5. Ion permeabilities for cASIC1a.

(A) Representative I-V relationships for cASIC1a in the presence of different cation ions with 140 mM KCl in the pipette. Currents were recorded at pH 6.0 using voltage ramps from -80 mV to +80 mV over 160 ms. (B) Relative ion permeabilities for PDPNaC1 and cASIC1a. p for K = 1.3×10^{-7}; p for Cs = 5.2×10^{-8}. Data information: In (B), data are presented as box-and-whisker plots showing median (center line), 25th and 75th percentiles (box limits), and minimum to maximum values (whiskers). All individual data points are shown, $n = 5$ cells for each group (Unpaired two-tailed Student's t-test).

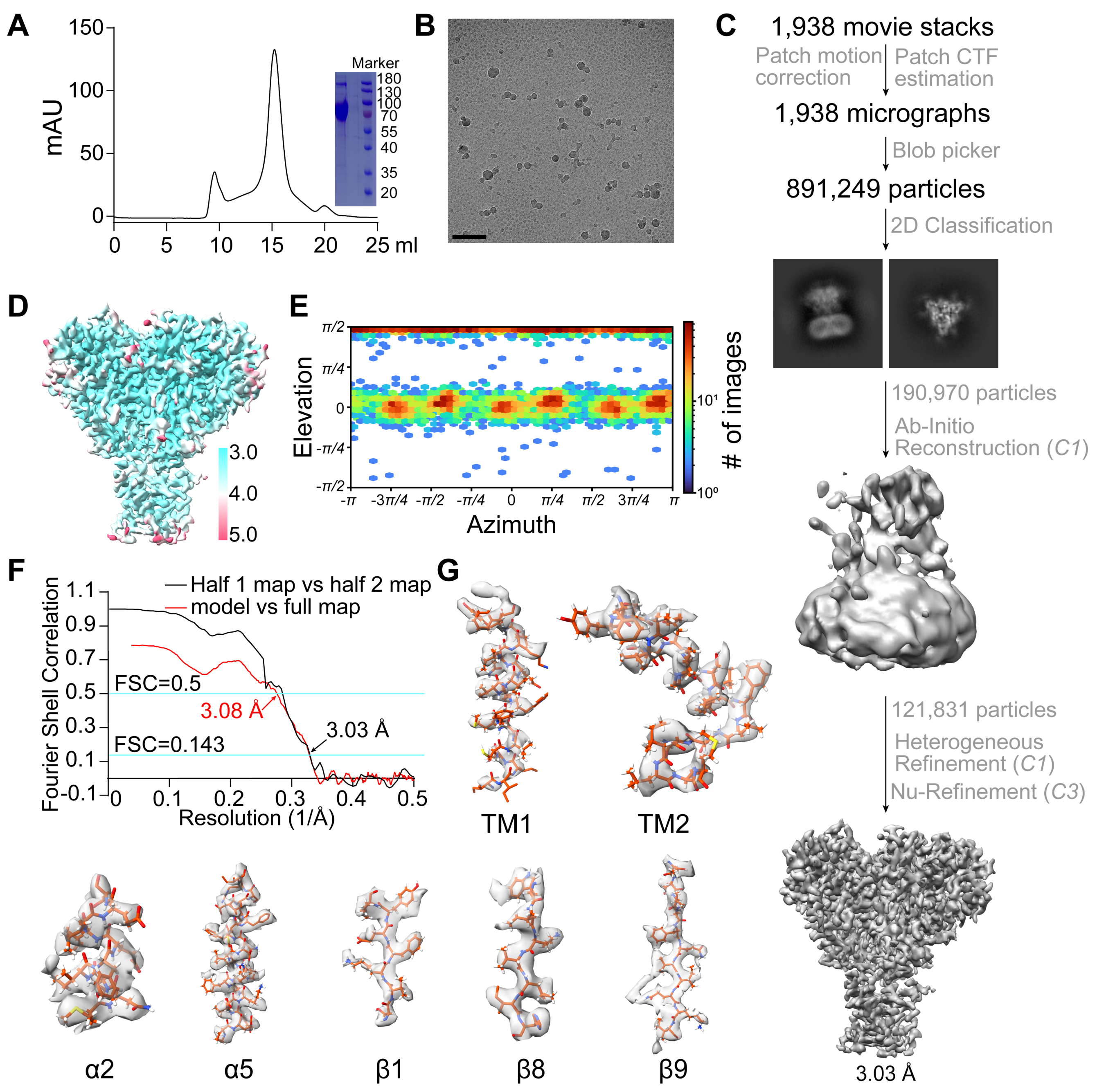

Appendix Figure S6. Protein collection and structure determination of PDPNaC1.

(A) Final size-exclusion chromatography (Superose 6, GE Healthcare) and SDS-PAGE analysis of PDPNaC1 samples.

(B) Representative cryo-EM micrograph of PDPNaC1 (scale bar: 100 nm).

(C) Flowchart of image processing.

(D) Final density map colored by local resolution.

(E) Distribution of orientations over azimuth and elevation angles for particles included in the calculation of the final map.

(F) Gold-standard Fourier Shell Correlation (FSC) curve of the final 3D reconstruction, and FSC curve for cross-validation between the map and the model.

(G) Sample density maps for various parts of the channel. The maps are low-pass filtered to 3.09 Å and sharpened with a B factor of -55.5 Å^2.

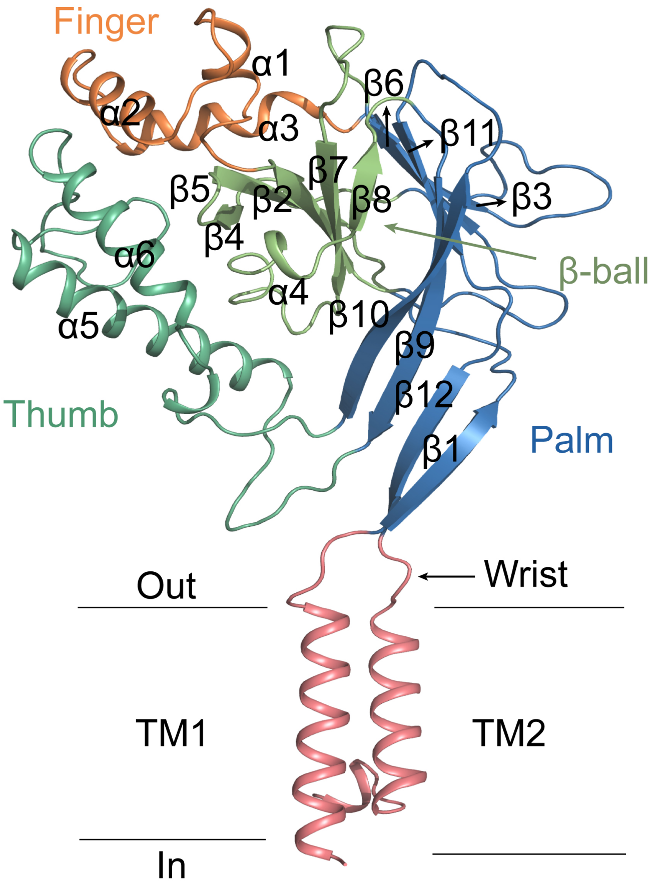

Appendix Figure S7. Ribbon diagram of a single subunit of PDPNaC1.
The secondary structural elements and domain organization are labeled.

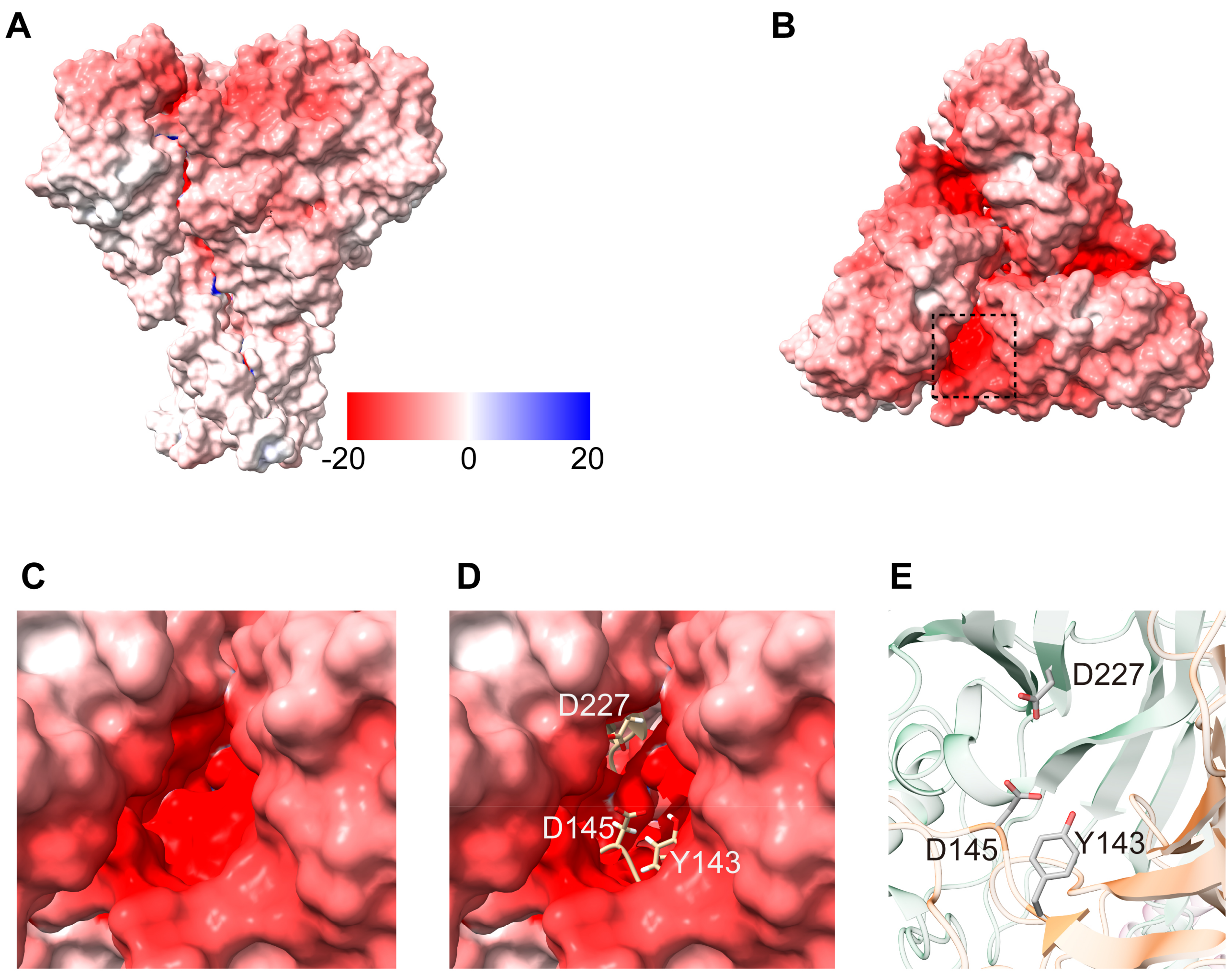

Appendix Figure. S8. Electrostatic potential map of PDPNaC1.

(A and B) Electrostatic potential mapped onto the solvent-accessible surface of the PDPNaC1 trimer highlights a highly negative cavity in the extracellular domain (boxed region). Contours are from -20 (red) to 20 kT (blue) through 0 (white).

(C) Focus view of the boxed region in panel B.

(D and E) Key residues in the acidic cavity outlined in panel C.

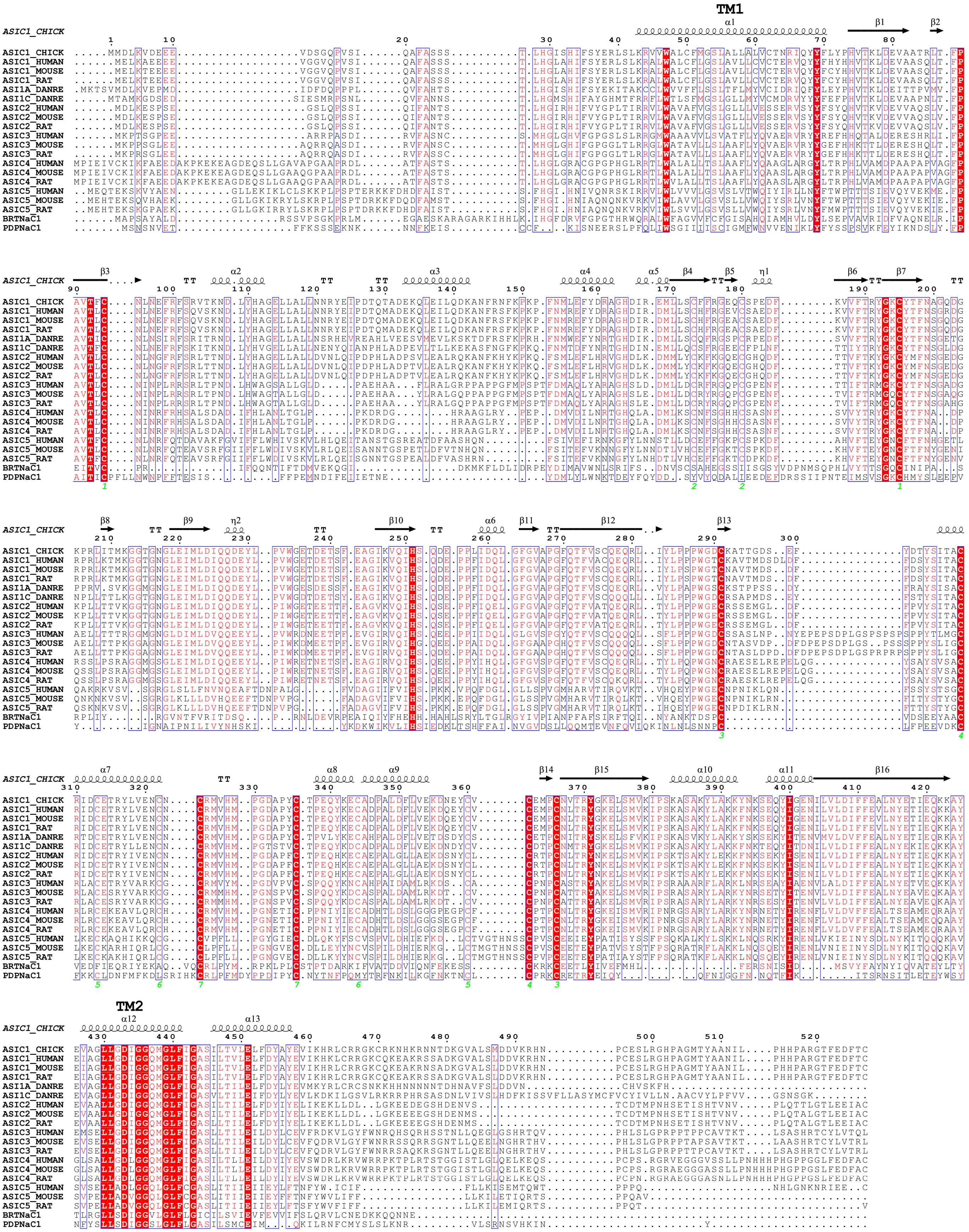

Appendix Figure S9. Sequence alignment.

Sequence alignment of amino acids between PDPNaC1 and ASIC channels was performed using MUSCLE, with visualization refined by ESPript (Easy Sequencing in Postscript) (for Gene IDs see Appendix Table S2). Secondary structure annotation of the sequence was performed based on the cryo-EM structure of cASIC1a (PDB: 5WKU).

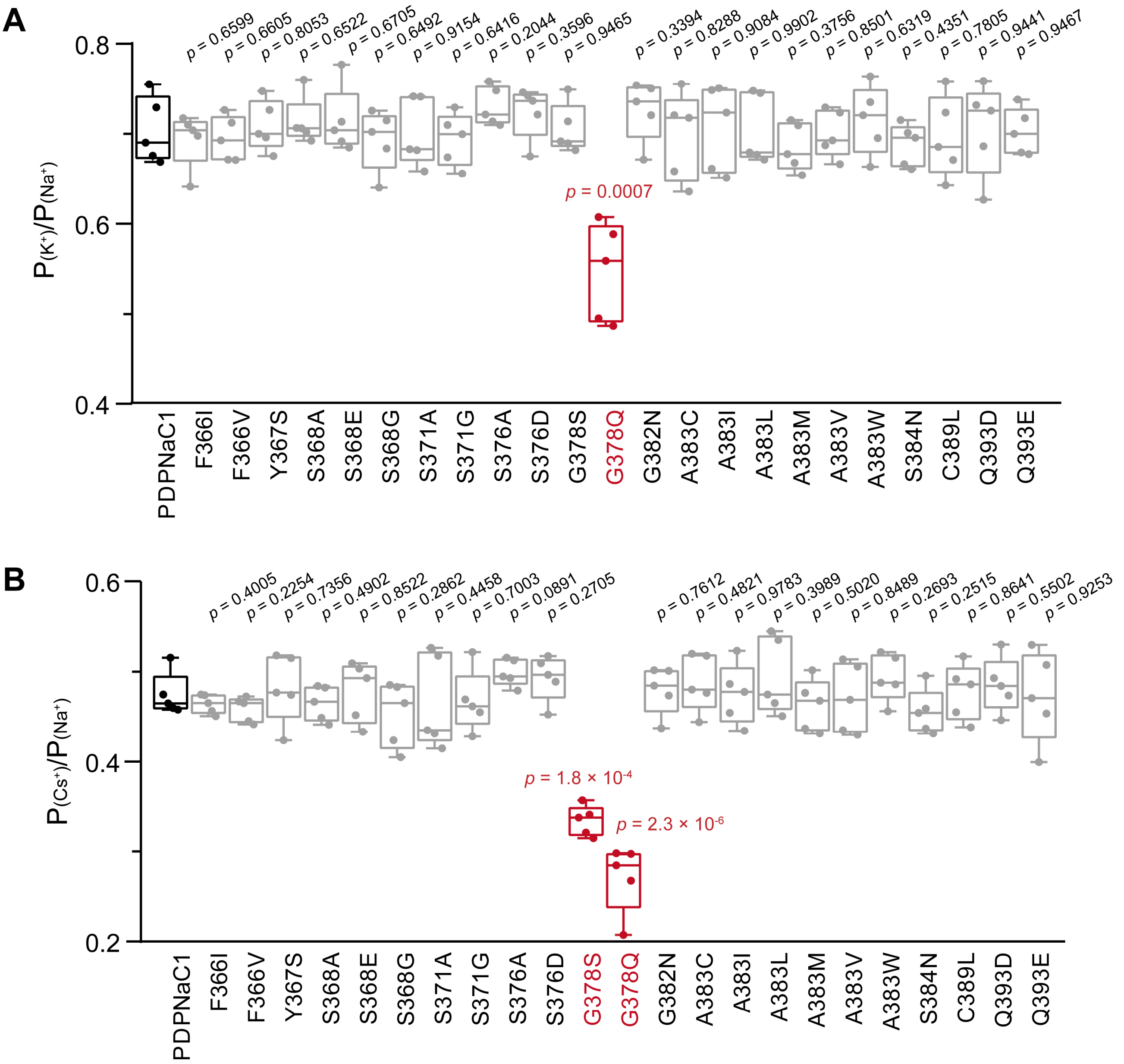

Appendix Figure S10. Ion selectivity of PDPNaC1 and mutants.

(A) Relative permeability ratio $P_{(K^+)}/P_{(Na^+)}$.

(B) Relative permeability ratio $P_{(Cs^+)}/P_{(Na^+)}$.

Data information: Data are presented as box-and-whisker plots showing median (center line), 25th and 75th percentiles (box limits), and minimum to maximum values (whiskers). All individual data points are shown, *n* = 5 cells for each group (Unpaired two-tailed Student's t-test).

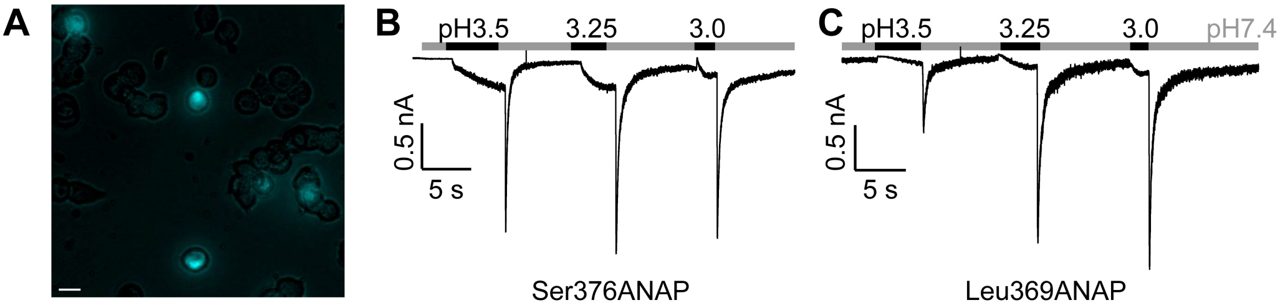

Appendix Figure S11. Functional analysis of ANAP-incorporated mutants.
(A) Representative images of cells expressing ANAP-incorporated PDPNaC1 in the presence of the pANAP vector. Scale bar: 20 µm.
(B and C) Representative current traces of Ser376ANAP-expressing (B) and Leu369ANAP-expressing (C) cells under different pH conditions (V_m = -80 mV).

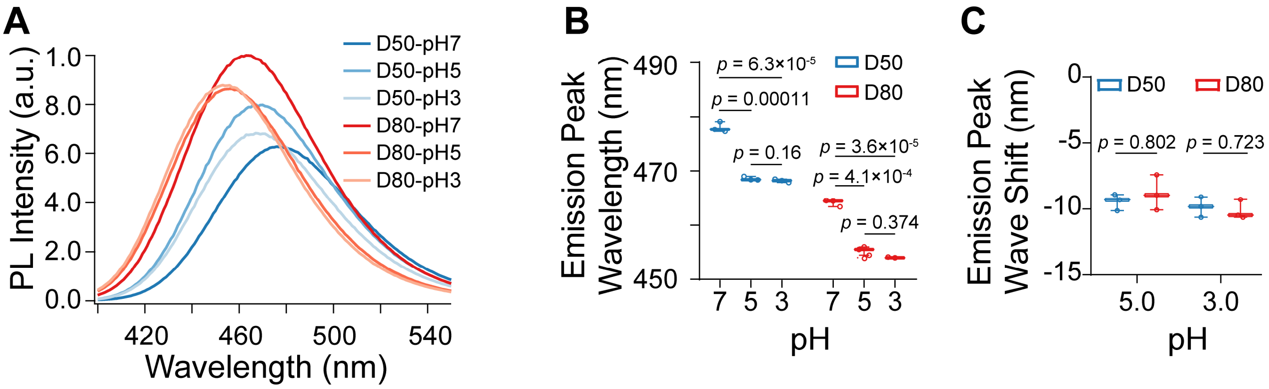

Appendix Figure. S12. Representative emission spectra of ANAP under different pH and DMSO conditions.

(A) Representative emission spectra of ANAP recorded in two solvent systems: 50% DMSO-50% PBS (D50) and 80% DMSO-20% PBS (D80), at pH 7.0, 5.0, and 3.0, respectively.

(B) Peak emission wavelengths extracted from the spectra shown in (A).

(C) Shift in peak emission wavelength at pH 5.0 and pH 3.0 relative to pH 7.0 in each solvent condition.

Data information: In (B and C), data are presented as box-and-whisker plots showing median (center line), 25th and 75th percentiles (box limits), and minimum to maximum values (whiskers). All individual data points are shown, $n = 3$ for each group (Unpaired two-tailed Student's t-test).

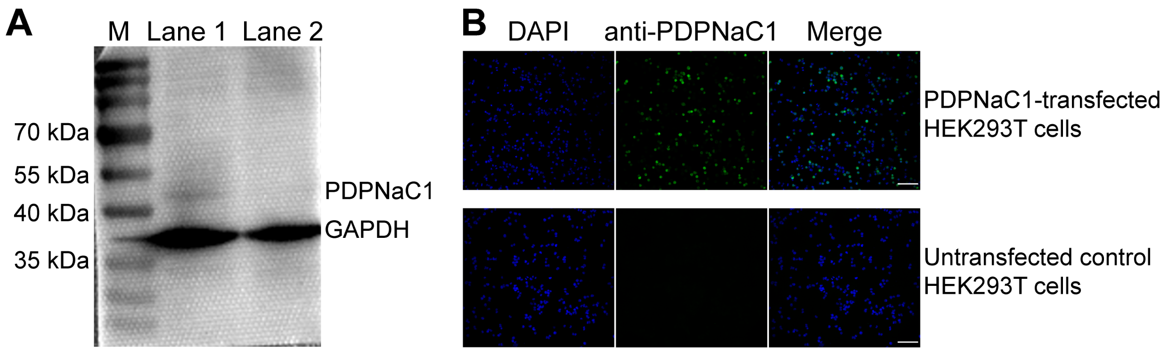

Appendix Figure S13. Anti-PDPNaC1 antibody validation by western blot and immunofluorescence in cultured cells.

(A) Western blot detection of PDPNaC1 (50 kDa) in HEK293T cells using an anti-PDPNaC1 antibody. Lane 1: PDPNaC1-transfected HEK293T cells; Lane 2: untransfected control. PDPNaC1 and GAPDH (36 kDa) were sequentially detected on the same membrane without stripping. M: molecular weight markers (kDa). The image corresponds to the second exposure after GAPDH detection. The original PDPNaC1 blot is provided in the Source Data.

(B) Immunofluorescence analysis of PDPNaC1 expression in HEK293T cells. HEK293T cells were transfected with PDPNaC1-expressing plasmids (top row) or untransfected as negative controls (bottom row). Confocal images show DAPI (left), PDPNaC1 signal (middle), and merged channels (right). Scale bar, 100 μm.

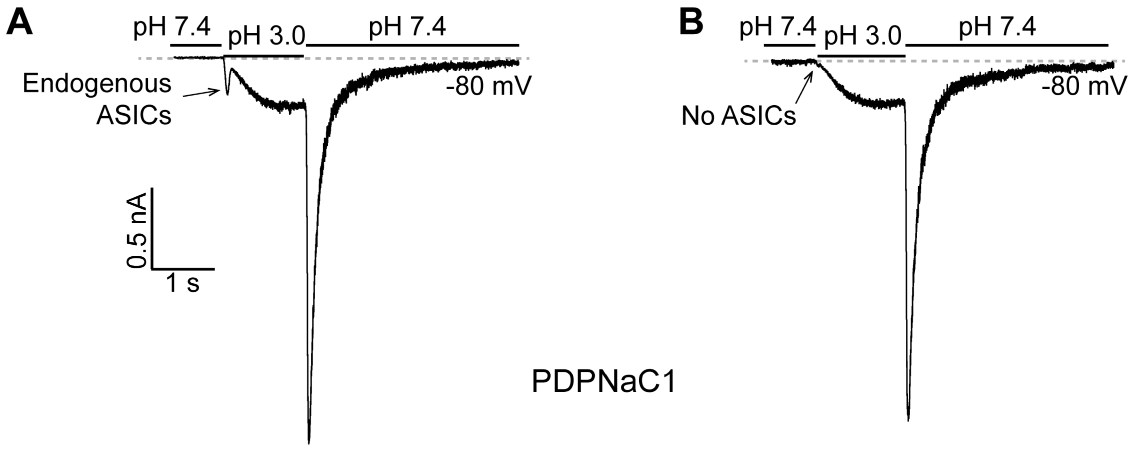

Appendix Figure S14. Representative current traces of PDPNaC1-expressing cells, showing the presence (A) or absence (B) of detectable endogenous ASIC currents.

Appendix Table S1. Cell number and annotation for each cell cluster and literature references for marker genes.

Cluster ID	Total number of cells	Cell type	Marker genes	Citation	PMID
0	4652	Neuron (unknown type)	*Ir25a*, *Acj6*, *Chat*, *Ano4*, *Gabbr2*, *Kcnip1*	Li H, et al. 2022; Task D, et al. 2022; Clyne PJ, et al. 1999; Shrestha BR, et al. 2018; Root CM, et al. 2008	35239393; 35442190; 10069339; 30078709; 18667158
3	1900	Neuron (unknown type)	*Ir25a, Acj6, Chat, Ano4, Gabbr2, Kcnip1*	Li H, et al. 2022; Task D, et al. 2022; Clyne PJ, et al. 1999; Shrestha BR, et al. 2018; Root CM, et al. 2008	35239393; 35442190; 10069339; 30078709; 18667158
4	982	Neuron (unknown type)	*Acj6, Chat, Slc32a1, Sox14*	Task D, et al. 2022; Clyne PJ, et al. 1999; Reiner BC, et al. 2021; Morello F, et al. 2020	35442190; 10069339; 34942400; 33053343; 35363520
2	2043	Metabolic cells	*Cyp2u1, Cyp4v2, Cyp2j2, Abcg1, Hsd17b13*	Li J, et al. 2016; Camp JG, et al. 2017; Zheng C, et al. 2017; Abul-Husn NS, et al. 2018	26691212; 28614297; 28622514; 29562163
5	976	Metabolic cells	*Cyp2u1, Cyp4v2, Cyp2j2, Abcg1, Hsd17b13*	Li J, et al. 2016; Camp JG, et al. 2017; Zheng C, et al. 2017; Abul-Husn NS, et al. 2018	26691212; 28614297; 28622514 29562163
10	841	Glia cells	*Eml1, Acox, Myrf, Cpe, Cryab*	Huang M, 2020; Tirosh I, 2016; Johnson TS, 2022	33028409; 27806376; 35105355
11	775	Immune cells	*Nlrc4, Mok, Lrp1, Cr2, Fancl, Pglyrp1*	Young MD, et al. 2018; Karamitros D, et al. 2018; Anand P, et al. 2021; Zhu R, et al. 2021; Petitprez F, et al. 2020; Kang D, et al. 1998	30093597; 29167569; 33227818; 34702946; 31942077; 9707603
6	912	Macrophages	*Mrc1, Mrc2*	Tu TH, et al. 2014; Li X, 2022	25477711; 32483412
7	891	Trachea-associated cells	*Abca3, P2rx4, Hephl1*	Xu Y, et al. 2016; Plasschaert LW, et al. 2018; Sharma P, et al. 2019	27942595; 30069046; 31125343
13	494	Muscle cells	*Mylk, Myl12b, Mlp60a, Ryr*	Guo M, 2015; Suizu F, 2000; Arber S, 1994; Sullivan KM, 2000	26600239; 10965042; 7954791; 10811919
14	411	Medullary cells	*Mag, Flt1, Sema5a*	Tang S, 1997; Adam M, 2017	9298990; 28851704
15	284	Pancreatic beta cells	*Pnliprp1, Rfx6*	Hendley AM, 2021; Muraro MJ, 2016	3400912; 27693023
1	4392	unknown type			
8	882	unknown type			
9	844	unknown type			
12	558	unknown type			

Appendix Table S2. List of proteins used for sequence alignment in this article.

Name	Protein	Database	Gene ID	Organism
BRTNaC1	BRTNaC1	GenBank	WCI13721.1	*Scolopendra mutilans*
PPK	pickpocket	Uniprot	Q7KT94	*Drosophila melanogaster*
PPK6	pickpocket 6	Uniprot	Q86LH3	*Drosophila melanogaster*
PPK7	pickpocket 7	Uniprot	Q9VME9	*Drosophila melanogaster*
PPK10	pickpocket 10	Uniprot	Q86LH1	*Drosophila melanogaster*
PPK11	pickpocket 11	Uniprot	Q9VL84	*Drosophila melanogaster*
PPK12	pickpocket 12	Uniprot	Q9W250	*Drosophila melanogaster*
PPK13	pickpocket 13	Uniprot	Q86LG9	*Drosophila melanogaster*
PPK14	Pickpocket 14	Uniprot	Q86LG8	*Drosophila melanogaster*
PPK15	Pickpocket 15	Uniprot	Q9VBF6	*Drosophila melanogaster*
PPK16	pickpocket 16	Uniprot	Q86LG7	*Drosophila melanogaster*
PPK17	pickpocket 17	Uniprot	Q9VJI4	*Drosophila melanogaster*
PPK18	Pickpocket 18	Uniprot	Q9VL88	*Drosophila melanogaster*
PPK19	pickpocket 19	Uniprot	Q9VAJ3	*Drosophila melanogaster*
PPK20	pickpocket 20	Uniprot	Q86LG5	*Drosophila melanogaster*
PPK21	pickpocket 21	Uniprot	Q86LG4	*Drosophila melanogaster*
PPK22	Pickpocket 22	Uniprot	Q8IMV2	*Drosophila melanogaster*
PPK23	pickpocket 23	Uniprot	M9PHF4	*Drosophila melanogaster*
PPK24	pickpocket 24	Uniprot	Q9V9Y5	*Drosophila melanogaster*
PPK25	pickpocket 25	Uniprot	A1Z6S4	*Drosophila melanogaster*
PPK26	pickpocket 26	Uniprot	Q9VS73	*Drosophila melanogaster*
PPK27	pickpocket 27	Uniprot	Q9VZN1	*Drosophila melanogaster*
PPK28	pickpocket 28	Uniprot	Q86LG1	*Drosophila melanogaster*
HyNaC2	Hydra Na channel 2	Uniprot	A8DZR6	*Hydra vulgaris*
HyNaC3	Hydra Na channel 3	Uniprot	A8DZR7	*Hydra vulgaris*
HyNaC4	Hydra Na channel 4	Uniprot	A8DZR8	*Hydra vulgaris*

HyNaC6	Hydra Na channel 6	Uniprot	A0A0A0MP54	*Hydra vulgaris*
HyNaC7	Hydra Na channel 7	Uniprot	A0A0A0MP73	*Hydra vulgaris*
HyNaC8	Hydra Na channel 8	Uniprot	A0A0A0MP67	*Hydra vulgaris*
HyNaC9	Hydra Na channel 9	Uniprot	A0A0A0MP48	*Hydra vulgaris*
HyNaC10	Hydra Na channel 10	Uniprot	A0A0A0MP61	*Hydra vulgaris*
HyNaC11	Hydra Na channel 11	Uniprot	A0A0A0MP55	*Hydra vulgaris*
HyNaC12	Hydra Na channel 12	Uniprot	A0A0A0MP74	*Hydra vulgaris*
MEC-10	MEC-10	Uniprot	P34886	*Caenorhabditis elegans*
MEC-4	MEC-4	Uniprot	P24612	*Caenorhabditis elegans*
MEC-2	MEC-2	Uniprot	Q27433	*Caenorhabditis elegans*
MEC-3	MEC-3	Uniprot	P09088	*Caenorhabditis elegans*
MEC-6	MEC-6	Uniprot	O01811	*Caenorhabditis elegans*
MEC-7	MEC-7	Uniprot	P12456	*Caenorhabditis elegans*
MEC-12	MEC-12	Uniprot	P91910	*Caenorhabditis elegans*
MEC-15	MEC-15	Uniprot	Q22071	*Caenorhabditis elegans*
MEC-17	MEC-17	Uniprot	O45100	*Caenorhabditis elegans*
DEG-1	DEG-1	Uniprot	P24585	*Caenorhabditis elegans*
DEL-1	DEL-1	Uniprot	Q19038	*Caenorhabditis elegans*
ASIC1_CHICK	ASIC1	Uniprot	Q1XA76	*Gallus gallus*
ASIC1_HUMAN	ASIC1	Uniprot	P78348	*Homo sapiens*
ASIC1_MOUSE	ASIC1	Uniprot	Q6NXK8	*Mus musculus*
ASIC1_RAT	ASIC1	Uniprot	P55926	*Rattus norvegicus*
ASI1A_DANRE	ASIC1	Uniprot	Q708S7	*Danio rerio*
ASI1C_DANRE	ASIC1	Uniprot	Q708S6	*Danio rerio*
ASIC2_HUMAN	ASIC2	Uniprot	Q16515	*Homo sapiens*
ASIC2_MOUSE	ASIC2	Uniprot	Q925H0	*Mus musculus*
ASIC2_RAT	ASIC2	Uniprot	Q62962	*Rattus norvegicus*
ASIC2_DANRE	ASIC2	Uniprot	Q708S5	*Danio rerio*
ASIC3_HUMAN	ASIC3	Uniprot	Q9UHC3	*Homo sapiens*
ASIC3_MOUSE	ASIC3	Uniprot	Q6X1Y6	*Mus musculus*
ASIC3_RAT	ASIC3	Uniprot	O35240	*Rattus norvegicus*
ASIC4_HUMAN	ASIC4	Uniprot	Q96FT7	*Homo sapiens*
ASIC4_MOUSE	ASIC4	Uniprot	Q7TNS7	*Mus musculus*
ASIC4_RAT	ASIC4	Uniprot	Q9JHS6	*Rattus norvegicus*
ASIC5_HUMAN	ASIC5	Uniprot	Q9NY37	*Homo sapiens*
ASIC5_MOUSE	ASIC5	Uniprot	Q9R0Y1	*Mus musculus*

ASIC5_RAT	ASIC5	Uniprot	Q9R0W5	*Rattus norvegicus*
AkFaNaC	FMRFamide-gated Na^+ channel	Uniprot	Q4H3X6	*Aplysia kurodai*
CaFaNaC	FMRFamide-activated amiloride-sensitive Na^+ channel	Uniprot	Q25011	*Cornu aspersum*
gammaENaC HUMAN	γENaC	Uniprot	P51170	*Homo sapiens*
betaENaC HUMAN	βENaC	Uniprot	P51168	*Homo sapiens*
alphaENaC HUMAN	αENaC	Uniprot	P37088	*Homo sapiens*
deltaENaC HUMAN	δENaC	Uniprot	P51172	*Homo sapiens*
alphaENaC CHICK	αENaC	Uniprot	Q92075	*Gallus gallus*
betaENaC SHEEP	βENaC	Uniprot	O62816	*Ovis aries*
alphaENaC CAVPO	αENaC	Uniprot	Q9R1N2	*Cavia porcellus*
alphaENaC BOVIN	αENaC	Uniprot	P55270	*Bos taurus*
gammaENaC MOUSE	γENaC	Uniprot	Q9WU39	*Mus musculus*
betaENaC MOUSE	βENaC	Uniprot	Q9WU38	*Mus musculus*
alphaENaC MOUSE	αENaC	Uniprot	Q61180	*Mus musculus*
alphaENaC RAT	αENaC	Uniprot	P37089	*Rattus norvegicus*
betaENaC RAT	βENaC	Uniprot	P37090	*Rattus norvegicus*
gammaENaC RAT	γENaC	Uniprot	P37091	*Rattus norvegicus*
betaENaC XENLA	βENaC	Uniprot	P51169	*Xenopus laevis*
alphaENaC XENLA	αENaC	Uniprot	P51167	*Xenopus laevis*
alphaENaC NEOFS	αENaC	Uniprot	H1AFJ5	*Neoceratodus forsteri*
betaENaC NEOFS	βENaC	Uniprot	H1AFJ6	*Neoceratodus forsteri*

Appendix Table S3. Primers used in this study to generate single-point mutants.

Mutants	Primer-Forward	Primer-Reverse
Phe366Ile	AGCTACAACATCTACAGCCTG CTGAGCGACATCG	AGGCTGTAGATGTTGTAGCTCCA GTACTCGGTCT
Phe366Val	AGCTACAACGTCTACAGCCTG CTGAGCGACATCG	AGGCTGTAGACGTTGTAGCTCCA GTACTCGGTCT
Tyr367Ser	CTACAACTTCTCCAGCCTGCT GAGCGACATCGGC	AGCAGGCTGGAGAAGTTGTAGC TCCAGTACTC
Ser368Ala	TACAACTTCTACGCCCTGCTG AGCGACATCGGCG	CTCAGCAGGGCGTAGAAGTTGT AGCTCCAGTACTCGG
Ser368Glu	AACTTCTACGAACTGCTGAGC GACATCGGCGGCA	TCAGCAGTTCGTAGAAGTTGTAG CTCCAGTACTCG
Ser368Gly	AACTTCTACGGCCTGCTGAGC GACATCGGCGGCA	TCAGCAGGCCGTAGAAGTTGTA GCTCCAGTACTCG
Ser371Ala	TACAGCCTGCTGGCCGACATC GGCGGCAGCCTGGGC	GCCGATGTCGGCCAGCAGGCTG TAGAAGTTGTAGCTC
Ser371Gly	ACAGCCTGCTGGGCGACATCG GCGGCAGCCTG	CGCCGATGTCGCCCAGCAGGCT GTAGAAGTTGT
Ser376Ala	ATCGGCGGCGCCCTGGGCCTG TTCCTGGGCGCCAGCA	AGGCCCAGGGCGCCGCCGATGT CGCTCAGCAGGCTGTA
Ser376Cys	TTCCTGGGCTGCAGCATCCTG AGCATGTGTGAGATCAT	TGCTCAGGATGCTGCAGCCCAG GAACAGGCCCAGGCT
Ser376Asp	ATCGGCGGCGACCTGGGCCTG TTCCTGGGCGCCA	CAGGCCCAGGTCGCCGCCGATG TCGCTCAGCAGGC
Gly378Gln	CGGCAGCCTGCAACTGTTCCT GGGCGCCAGCATC	CAGGAACAGTTGCAGGCTGCCG CCGATGTCGCTCA
Gly378Ser	CGGCAGCCTGAGCCTGTTCCT GGGCGCCAGCATC	CAGGAACAGGCTCAGGCTGCCG CCGATGTCGCTCA
Gly382Asn	CTGTTCCTGAACGCCAGCATC CTGAGCATGTGTGAGAT	ATGCTGGCGTTCAGGAACAGGC CCAGGCTGCCGCCGA
Ala383Cys	TTCCTGGGCTGCAGCATCCTG AGCATGTGTGAGATCAT	TGCTCAGGATGCTGCAGCCCAG GAACAGGCCCAGGCT
Ala383Ile	TTCCTGGGCATCAGCATCCTG AGCATGTGTGAGATCAT	TGCTCAGGATGCTGATGCCCAGG AACAGGCCCAGGCT
Ala383Leu	TTCCTGGGCCTCAGCATCCTG AGCATGTGTGAGATCAT	TGCTCAGGATGCTGAGGCCCAG GAACAGGCCCAGGCT
Ala383Met	TTCCTGGGCATGAGCATCCTG AGCATGTGTGAGATCAT	TGCTCAGGATGCTCATGCCCAGG AACAGGCCCAGGCT
Ala383Val	TTCCTGGGCGTCAGCATCCTG AGCATGTGTGAGATCAT	TGCTCAGGATGCTGACGCCCAG GAACAGGCCCAGGCT
Ala383Trp	TTCCTGGGCTGGAGCATCCTG AGCATGTGTGAGATCAT	TGCTCAGGATGCTCCAGCCCAG GAACAGGCCCAGGCT
Ala384Asn	CTGGGCGCCAACATCCTGAGC ATGTGTGAGATCAT	TGCTCAGGATGTTGGCGCCCAG GAACAGGCCCAGG
Cys389Ler	CCTGAGCATGCTTGAGATCAT GCAGAAGATCCTGAG	CATGATCTCAAGCATGCTCAGGA TGCTGGCGCC
Gln393Asp	GAGATCATGGACAAGATCCTG AGAAACTTCTGCA	TTCTCAGGATCTTGTCCATGATC TCACACATGCTCAGGA
Gln393Glu	AGATCATGGAGAAGATCCTGA GAAACTTCTGCATGTAC	TCAGGATCTTCTCCATGATCTCA CACATGCTCAGGAT
Leu369ANAP	CTTCTACAGCTAGCTGAGCGA CATCGGCGGCAGC	ATGTCGCTCAGCTAGCTGTAGAA GTTGTAGCTCCAG
Ser376ANAP	CATCGGCGGCTAGCTGGGCCT GTTCCTGGGCGCCA	CAGGCCCAGCTAGCCGCCGATG TCGCTCAGCAGG

Appendix Table S4. Data collection and refinement statistics.

**Data collection and processing**	**PDPNaC1**
Microscope	FEI Titan Krios
Voltage(keV)	300
Camera	Falcon4 detector
Magnification	140,000×
Electron exposure (e^-/Å^2)	~52
Defocus range (μm)	–0.8 to –1.6
Pixel size (Å)	0.93
Symmetry imposed	*C3*
Micrographs (no.)	1,938
Initial particle images (no.)	891,249
Final particle images (no.)	121,831
Map resolution (Å)	3.03
FSC threshold	0.143
Refinement	
Initial model used (PDB code)	*de novo*
Model resolution (Å)	3.08
FSC threshold	0.5
Map sharpening B factor (Å^2)	-55.5
Model composition	
Non-hydrogen atoms	8,592
Protein residues	1,032
Ligands	0
B factors (Å^2)	
Protein	57
Ligand	
R.m.s. deviations	
Bond lengths (Å)	0.006
Bond angles (°)	1.340
Validation	
MolProbity score	2.68
Clashscore	17.75
Rotamer outliers (%)	0
Ramachandran plot	
Favored (%)	95.61
Allowed (%)	4.39
Disallowed (%)	0
